# Photonic Bayesian Neural Networks: Leveraging Programmable Noise for Robust and Uncertainty‐Aware Computing

**DOI:** 10.1002/advs.202500525

**Published:** 2025-04-27

**Authors:** Yangyang Zhuge, Zhihao Ren, Zian Xiao, Zixuan Zhang, Xinmiao Liu, Weixin Liu, Siyu Xu, Chong Pei Ho, Nanxi Li, Chengkuo Lee

**Affiliations:** ^1^ Department of Electrical and Computer Engineering National University of Singapore 4 Engineering Drive 3 Singapore 117583 Singapore; ^2^ Center for Intelligent Sensors and MEMS National University of Singapore 4 Engineering Drive 3 Singapore 117583 Singapore; ^3^ Institute of Microelectronics (IME) Agency for Science Technology and Research (A*STAR) 2 Fusionopolis Way, Innovis #08‐02 Singapore 138634 Singapore; ^4^ National Centre for Advanced Integrated Photonics (NCAIP) National University of Singapore Singapore 119077 Singapore; ^5^ NUS Suzhou Research Institute (NUSRI) Suzhou Jiangsu 215123 China; ^6^ NUS Graduate School‐Integrative Sciences and Engineering Programme (ISEP) National University of Singapore Singapore 119077 Singapore

**Keywords:** Bayesian neural network, multimodal data processing, photonic probabilistic computing, photonic random number generator, silicon photonics

## Abstract

Photonic neural networks (PNNs) based on silicon photonic integrated circuits (Si‐PICs) offer significant advantages over microelectronic counterparts, including lower energy consumption, higher bandwidth, and faster computing speeds. However, the analog nature of optical signal in PNNs makes Si‐PIC solutions highly sensitive to device noise, especially when using fixed‐value deterministic models, which are not robust to hardware fluctuation. Furthermore, current PNNs are unable to handle data uncertainty, a critical factor in applications such as autonomous driving, medical diagnostics, and financial forecasting. Herein, a photonic Bayesian neural network (PBNN) architecture that incorporates Bayesian principles to enhance robustness and address uncertainty is proposed. In the PBNN, device noise is leveraged through photonic‐noise‐based random number generators, which combine Mach‐Zehnder interferometers and micro‐ring resonators to independently control output mean and standard deviation. Based on modelling with experimentally extracted data, the PBNN achieves a classification accuracy of up to 98% for handwritten digit recognition, matching full‐precision models on conventional computers. Beyond classification, the PBNN excels in multimodal data processing, regression, and outlier detection. This scalable, energy‐efficient architecture transforms photonic noise into computational value, addressing the limitations of deterministic PNNs and enabling uncertainty‐aware computing for real‐world applications.

## Graphical abstract

This work introduces a novel photonic Bayesian neural network architecture that utilizes tunable photonic random number generators to independently control weight distribution parameters, significantly improving robustness, scalability, and accuracy while demonstrating practical applications in multimodal data processing and uncertainty‐aware computing.

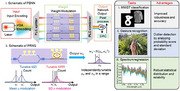

## 1 Introduction

With the rapid advancement of artificial neural network algorithms in fields such as pattern recognition and image classification,^[^ ^1^, ^2^ ^]^ the design of supporting hardware architectures has emerged as a significant research area. Beyond traditional electronic architectures based on transistors and memristors,^[^ ^3^, ^4^ ^]^ photonic neural networks (PNNs) have gained considerable attention due to advances in photonic integrated circuit technology. ^[^ ^5^, ^6^ ^]^ PNNs leverage light to transmit data and perform matrix multiplications using photonic devices. Compared to their electronic counterparts, PNNs offer advantages such as low loss and reduced energy consumption on the silicon‐on‐insulator (SOI) platform.^[^ ^7^, ^8^ ^]^ Additionally, by fully utilizing wavelength division multiplexing (WDM) technology, PNNs enable the parallel processing of multi‐channel data, paving the way for high‐speed, energy‐efficient computing.^[^ ^9^, ^10^, ^11^ ^]^

**Figure 1:**
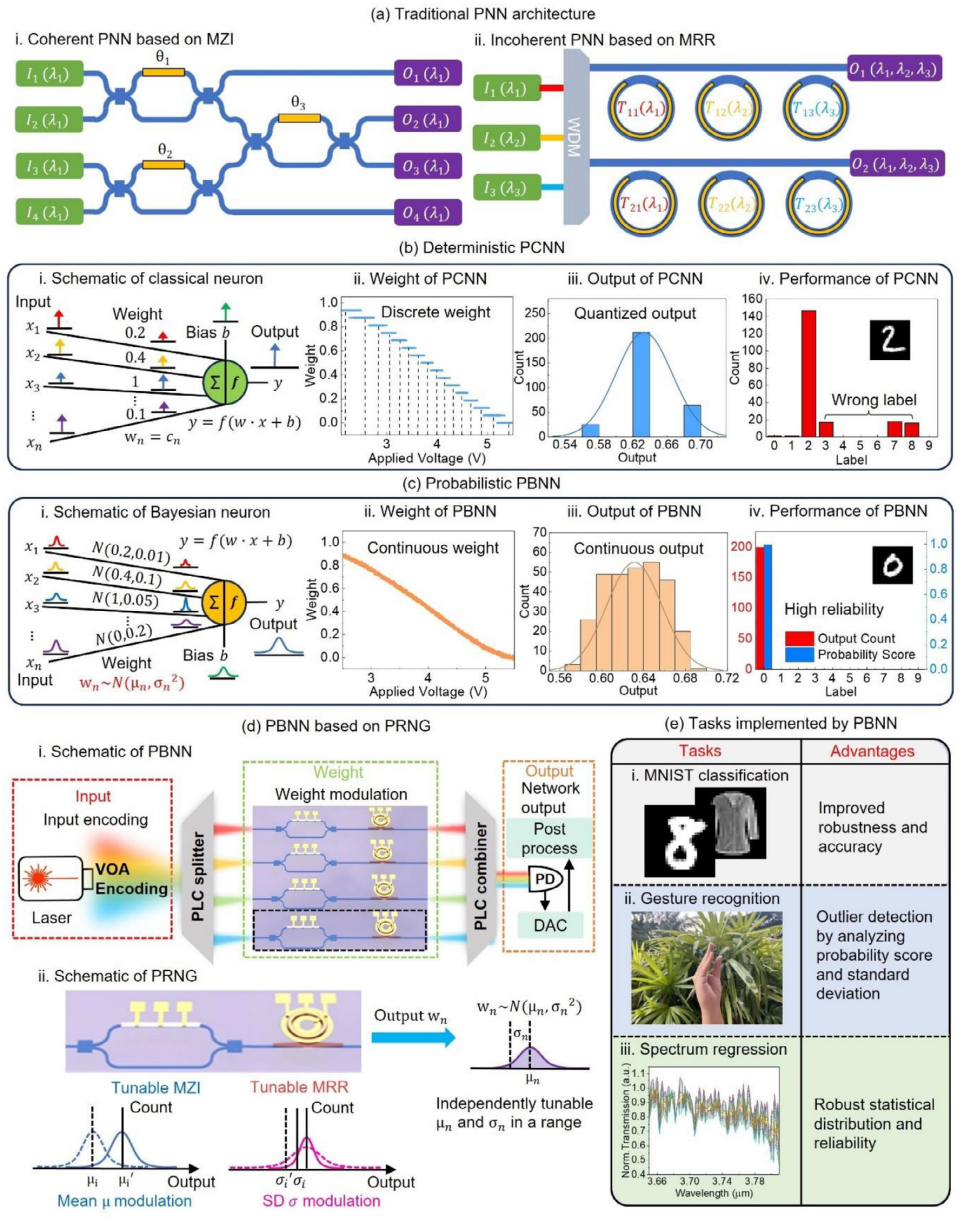
Comparison between PCNN and PBNN. a) Traditional PNN architecture. i) Coherent PNN based on an MZI array. ii. Incoherent PNN based on an MRR weight bank. b) Deterministic PCNN. i) Schematic of a classical neuron used in PCNN. ii) Weight control in PCNN, which uses quantization to reduce noise impact. iii) Output distribution of a neuron in PCNN, showing unstable quantized outputs affected by noise. iv) PCNN performance on the MNIST task, demonstrating output fluctuation due to noise. c) Probabilistic PBNN. i) Schematic of a Bayesian neuron used in PBNN. ii) Weight control in PBNN, utilizing noisy, continuous weights to improve robustness. iii) Output distribution of a neuron in PBNN, which follows a Gaussian distribution. iv) PBNN performance on the MNIST task, showing improved robustness to noise, and probability scores indicating reliability. d) PBNN based on PRNG. Light intensities encoded by VOAs serve as PBNN inputs. The PRNG, composed of tunable MZIs and MRRs, adjusts output intensities as weights. The MZI modulates the weight mean, while the MRR controls the standard deviation (SD). Outputs are detected by photodetectors (PDs) and processed on the personal computer (PC). e) PBNN applications. The Bayesian approach enhances network robustness, enables outlier detection, and provides probabilistic predictions.

Current PNN architectures are broadly classified into coherent and incoherent types, as shown in **Figure** **1**a. To achieve matrix‐vector multiplication (MVM) in neural network layers, coherent PNNs primarily utilize Mach‐Zehnder interferometer (MZI) array^[^ ^12^ ^]^ or diffractive spatial device,^[^ ^8^ ^]^ whereas incoherent PNNs include WDM crossbar array and micro‐ring resonator (MRR) weight bank.^[^ ^13^ ^]^ Both architectures rely on modulators to program photonic devices and mimic the MVM operations. However, since optical signals are inherently analog and most current PNNs use thermo‐optic effect‐based modulators, their outputs are highly sensitive to deviations caused by crosstalk or limited precision in the modulation voltage.^[^ ^6^, ^7^ ^]^ Furthermore, most reported PNNs employ deterministic models, such as multilayer perceptron (MLP) and convolutional neural network, to perform artificial intelligence (AI) computing tasks. In these networks, each neuron—including input, weight, bias, and output—is represented by fixed, high‐precision value, as depicted in Figure 1b‐i. The deterministic nature of these models renders them vulnerable to noise and fluctuations. As a result, even minor errors in neuron outputs can accumulate and significantly reduce network accuracy.^[^ ^14^ ^]^

**Figure 2:**
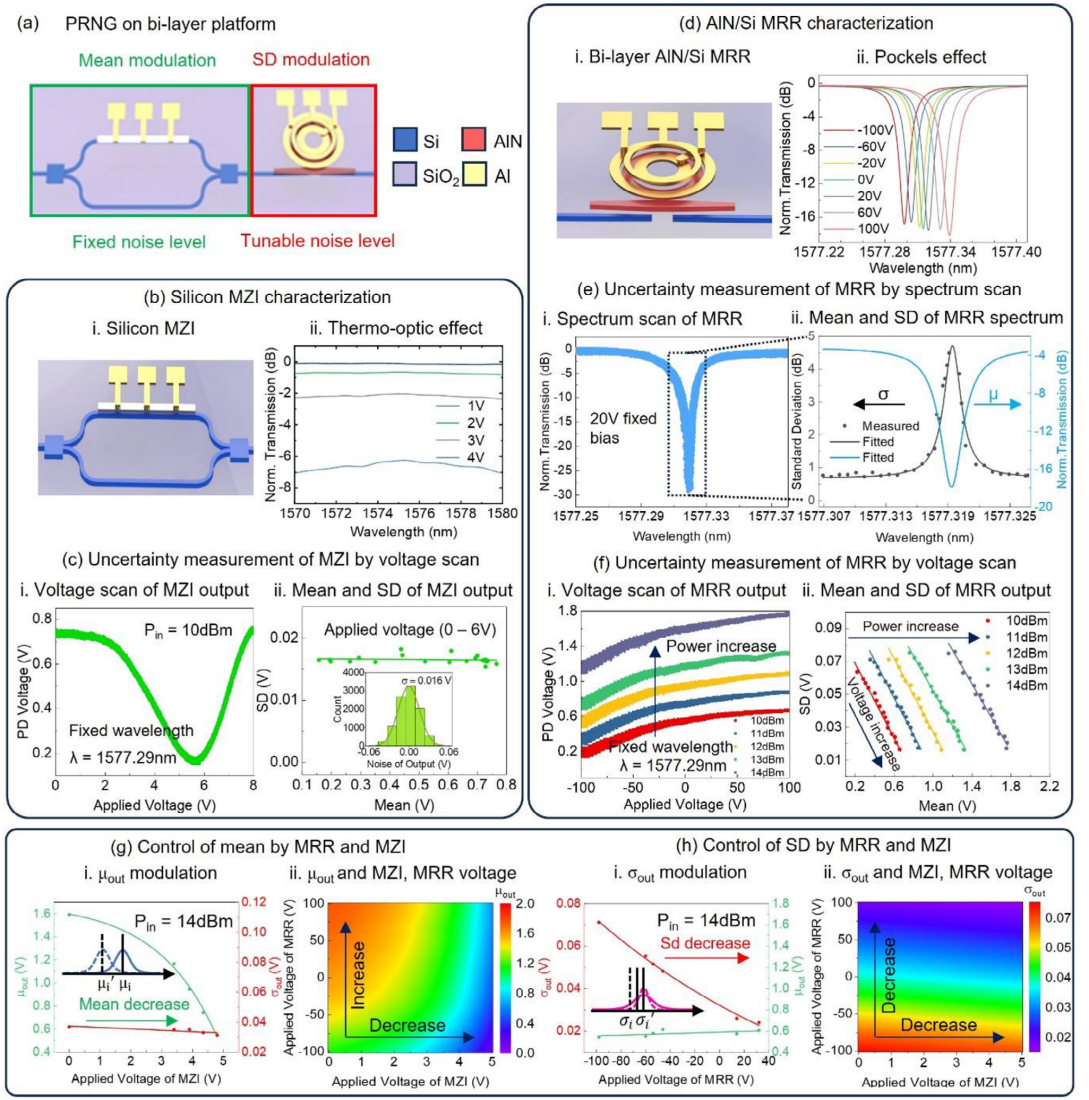
Implementation of PRNG by combining MZI and MRR. a) Schematic of the PRNG unit based on bi‐layer structure. b) Characterization of the Silicon MZI. i) Schematic of the silicon thermo‐optic MZI. ii. Measured MZI spectrum at various applied voltages. c) Uncertainty measurement of the MZI via voltage scan. i) Output PD voltage of the MZI at 10 dBm input power under various applied voltages. ii) Fitted mean and standard deviation of the MZI output. d) Characterization of the AlN/Si MRR. i) Schematic of the bi‐layer AlN/Si MRR. ii) Spectrum modulation of the MRR through the Pockels effect in AlN. e) Uncertainty measurement of the MRR via spectrum scan. i) Spectrum results from 500 measurements. ii) Fitted mean and standard deviation of transmission at different wavelengths. f) Uncertainty measurement of the MRR via voltage scan. i) Output PD voltage under various applied voltages and input powers. ii. Fitted mean and standard deviation of the 5 curves under different input powers in i. g) Control of PRNG output mean (*μ_out_*). i) Modulation of *μ_out_*. The mean of weight decreases as the applied voltage on the MZI increases. ii. Relationship between *μ_out_* and MZI/MRR voltages. h) Control of PRNG output standard deviation (*σ_out_*). i) Modulation of *σ_out_*. The standard deviation decreases as the MRR voltage increases, while a nearly unchanged mean output is maintained by adjusting the applied voltage of MZI. ii) Relationship between *σ_out_* ​ and MZI/MRR voltages.

Various approaches have been proposed to mitigate the impact of device noise, including output calibration and weight quantization, but these methods increase system complexity and provide limited improvements.^[^ ^15^, ^16^, ^17^ ^]^ Figure 1b‐ii presents experimental results of 4‐bit quantized outputs from a balanced Si MZI tuned by TiN microheater on top (further details are available in **Figure** **2** and Note S1 Supporting Information). Although weight quantization reduces the effects of device noise and stabilizes outputs, it also degrades the inherent accuracy of the neural network due to model compression.^[^ ^18^ ^]^ Moreover, applying ≈3.5 V to the device in Figure 1b‐ii demonstrates the remaining possibility of incorrect outputs, as illustrated in Figure 1b‐iii. Consequently, in Figure 1b‐iv, the outputs of deterministic photonic classical neural network (PCNN) are still uncertain, even for well‐trained models implemented on an electronic processor.^[^ ^19^ ^]^ In addition to device uncertainty, deterministic neural networks cannot address data uncertainty, which is critical in applications such as autonomous driving and medical diagnostics, where machine learning models must incorporate uncertainty‐aware mechanisms or facilitate human oversight.^[^ ^20^, ^21^ ^]^ While the deterministic models are prone to overfitting and frequently produce overly confident predictions.

The challenges of noise robustness and uncertainty analysis can be effectively addressed by incorporating Bayesian neural network (BNN) algorithms into PNNs.^[^ ^22^, ^23^ ^]^ In BNNs, as shown in Figure 1c‐i, weights and biases follow probability distributions, enabling output fluctuation and uncertainty characterization.^[^ ^24^ ^]^ Consequently, in PBNNs, the noisy outputs of optical devices (as depicted in Figure 1c‐ii,c‐iii) can directly serve as Bayesian neurons’ outputs, leveraging the analog nature of optical signals. Here we also take the results of the MZI in Figure 1b as an example to briefly demonstrate how the noise‐influenced optical output can be converted into a continuous Bayesian weight with a specific probability distribution. By accounting for uncertainty, PBNNs produce stable and accurate predictions despite device noise and fluctuations.^[^ ^25^, ^26^ ^]^ Moreover, the statistical nature of BNNs allows PBNNs to provide uncertainty information, reflected by output distributions. For instance, an output with a high probability score and minor fluctuation, as shown in Figure 1c‐iv, typically indicates a high confidence level in the PBNN's prediction.

The distinction between BNNs and deterministic models lies in the representation of weights. In deterministic models, weights are fixed values obtained by continuously fitting the certain input‐output relationship, so they must have high precision and no deviation. In contrast, BNNs represent weights as probability distributions, allowing them to fluctuate and capture uncertainty in the probabilistic model. Thus, the critical component of BNN hardware implementations is the realization of random number generators (RNGs) capable of generating random outputs with tunable distributions.^[^ ^27^ ^]^ In BNN enabled by electronic processors, RNGs exploit the inherent randomness of the memristor switching process. However, this mechanism introduces significant complexity and prolongs the computation time, as output distributions must be captured through repeated memristor read‐and‐write cycles. Additionally, physical constraints often enforce fixed relationships between the mean (μ) and standard deviation (σ) of device outputs. For instance, variations in modulation condition or device material properties simultaneously affect both μ and σ, creating a fixed, non‐tunable relationship between these parameters. This coupling restricts the flexibility of the RNG in generating diverse probabilistic distributions needed for Bayesian inference. To address this, researchers combine two types of devices with differing μ‐σ relationships to function as an RNG for BNNs.^[^ ^28^, ^29^, ^30^, ^31^ ^]^ This dual‐device system provides greater flexibility by allowing the synthesis of a wider range of output distributions. However, it also increases hardware complexity and resource usage, making it less ideal for scalable and energy‐efficient implementations of BNNs. These challenges underscore the need for alternative solutions, such as photonic‐based RNGs, which offer high‐speed, low‐energy randomness generation enabled by the analog nature of optical signal.

Research on photonic implementations of BNNs remains limited, focusing primarily on achieving probabilistic computing for parallel processing, simplifying readout and sampling processes. Wu et al. and Frank et al. proposed PBNNs utilizing amplified spontaneous emission (ASE) to generate random outputs.^[^ ^25^, ^26^, ^27^ ^]^ Since ASE noise level is also related to signal intensity, Wu et al. used the same MRR weight bank setup twice to compute mean and standard deviation separately, but their work focused more on conceptual development and lacked sufficient experimental data.^[^ ^25^ ^]^ Frank et al. independently controlled mean and standard deviation by sequentially summing ASE signals of varying intensities.^[^ ^26^ ^]^ In the ASE‐enabled architecture, the random distribution is modulated by the input light source, limiting its flexibility. Phase change material cells, which serve as weight elements, provide fixed attenuation ratios but lack the ability to independently tune μ and σ of the output distribution. These limitations restrict the number of tunable distributions that can be generated, reducing the architecture's adaptability and scalability for probabilistic computations. Consequently, this structure is primarily suited for Bayesian convolution layers, where fewer tunable distributions are sufficient, rather than for fully connected layers, which require greater flexibility in modelling complex probabilistic relationships.

In this study, we propose an integrated PBNN architecture that leverages photonic‐noise‐based photonic random number generators (PRNGs), as shown in Figure 1d. Unlike previous ASE‐based PRNGs, our design exploits the output uncertainty of photonic devices induced by fluctuations in the driving voltage. To address the fixed relationship between the μ and σ inherent in other designs, we combine an MZI and an MRR together to work as a PRNG, allowing independent control over the μ and σ of the unit. The MRR, characterized by its output‐dependent and broad‐range noise levels, modulates σ by leveraging its steep spectral response at resonance. In contrast, the balanced MZI, with output‐independent and moderate noise levels, tunes μ correspondingly based on the MRR output. This complementary configuration provides flexible control over the probabilistic characteristics of the PRNG. Following comprehensive device characterization, each PRNG can be calibrated to function as a Bayesian weight with tunable μ and σ. This tunability enables the integration of PRNGs into large‐scale Bayesian fully connected layers, significantly enhancing the capacity of PBNNs to perform robust probabilistic computations at scale. By facilitating flexible, high‐speed, and energy‐efficient Bayesian inference, this design represents a critical advancement in uncertainty‐aware photonic computing (Figure 1e).

For basic handwritten digit classification tasks, the PBNN achieves high classification accuracy and robustness to device noise, showcasing the fundamental benefits of the BNN algorithm on photonic hardware platforms.^[^ ^32^ ^]^ Furthermore, for multimodal datasets containing diverse data forms and uncertainties,^[^ ^33^ ^]^ the statistical nature of BNNs enables effective feature extraction from the complex and uncertain data. This capability allows the PBNN to achieve high classification accuracy on expected inputs while distinguishing outliers with possible outcomes based on probability. The inherent uncertainty modelling of BNN further supports reliable regression and mitigates overfitting, enhancing performance across various tasks.

## 2 Photonic Random Number Generator

The PRNG is a foundational component of PBNN because it generates the random values necessary for Bayesian inference, enabling the modelling of uncertainty in weights and data. By providing tunable probabilistic outputs and leveraging photonic device noise, the PRNG ensures efficient, scalable, and robust probabilistic computations critical for PBNN performance. Due to the inability to independently adjust μ and σ using only an MZI or an MRR, we employ a series connection of an MZI and an MRR to function as a PRNG, as shown in **Figure** **2**a. The PRNG is fabricated using bilayer waveguides, consisting of a silicon bottom layer and an AlN top layer, achieved through a monolithic integration process (Note S2, Supporting Information). The MZI, composed of silicon waveguides, takes advantage of silicon's significant thermo‐optic effect, while the MRR, comprising an AlN ring and silicon waveguides, utilizes AlN's electro‐optic effect and thermal stability.^[^ ^34^, ^35^, ^36^, ^37^ ^]^ This complementary use of Si and AlN materials enhances the PRNG's tunability and performance, enabling precise and efficient probabilistic computations for PBNN.

In the MZI shown in Figure 2b‐i, a TiN heater locally heats one arm, inducing a refractive index change via the thermo‐optic effect. This introduces a phase difference between the two arms, modulating the optical intensity at the output port through constructive or destructive interference. The modulated spectra, measured under varying DC voltages, are shown in Figure 2b‐ii, demonstrating a relatively flat spectral response. Categorizing output noise sources into intensity noise (e.g., photodetector noise, light source fluctuations, and fiber‐to‐chip coupling variations) and wavelength noise (e.g., light source wavelength shifts and refractive index fluctuations),^[^ ^38^ ^]^ the broadband modulation of the MZI output reduces susceptibility to wavelength noise.

To characterize the MZI's output uncertainty, a voltage scan method was employed. The input laser wavelength was fixed while the output intensity was measured across varying voltages. Due to the influence of electrical noise, the modulation voltage of the device fluctuates within a certain range, leading to variations in the refractive index and spectral response. These fluctuations are ultimately manifested as noise in the optical output. Figure 2c‐i shows the noisy outputs of the MZI under different applied voltages, revealing stable, output‐independent noise levels. Because electrical noise—such as thermal noise and shot noise—approximately follows Gaussian distributions in the time domain, the optical output noise induced by such electrical fluctuations can also be well modeled using Gaussian distributions. This is demonstrated in results of the main text, as well as Notes S3 and S4 (Supporting Information), where experimental results show good agreement between the output distributions and Gaussian fits. By performing multiple samplings of the output under specific modulation conditions, we can extract the mean and standard deviation of the output through Gaussian fitting of the resulting histograms. Figure 2c‐ii depicts the relationship between the fitted mean and standard deviation, with a stable standard deviation of ≈0.016 V. The histogram of all output fluctuation in the subgraph of Figure 2c‐ii confirms an output‐independent noise level, with a fitted mean of 0 V and a standard deviation of 0.016 V.

In the MRR depicted in Figure 2d‐i, 60 µm long adiabatic couplers couples light between the silicon and AlN layers with a coupling loss of 0.06 dB per transition. The AlN ring enables modulation via the Pockels effect. The MRR spectra under different applied voltages are shown in Figure 2d‐ii, demonstrating a resonance peak tunability of 0.26 pm/V. Unlike the MZI, the MRR exhibits significant transmission changes near its resonant wavelength due to its resonance cavity properties, where even a 5 pm wavelength shift can cause a transmission change exceeding 10 dB. This makes the MRR highly sensitive to wavelength noise near resonance, although it is commonly operated in this region for effective modulation. To characterize MRR uncertainty, two methods were used: a voltage scan (similar to the method for MZI) and a spectrum scan. In the spectrum scan, the applied voltage was fixed while the spectrum was scanned repeatedly. Figure 2e‐i displays spectrum scan results, showing significant fluctuations near the resonant wavelength and minor disturbances elsewhere. Figure 2e‐ii plots the fitted mean and standard deviation of transmission around the resonance peak, where the average output is inversely proportional to the standard deviation, consisting with the MRR's all‐pass structure characteristic.^[^ ^39^ ^]^For the voltage scan, both applied voltage and input power (10–14 dBm) were varied, as shown in Figure 2f‐i. The results align with the spectral scanning method, showing decreased output and increased noise levels as the laser wavelength approaches the resonant wavelength. Noise levels at 0 V were not minimal, indicating that spectrum shifts due to voltage fluctuations are the primary noise source, independent of voltage magnitude. Figure 2f‐ii shows the MRR output's fitted mean and standard deviation across varying input powers, with means ranging from ≈0.2 to 1.8 V and standard deviations from ≈0.015 to 0.08 V. The MZI and MRR results indicate that spectrum shifts from voltage fluctuations are the dominant noise source. A control experiment with constant laser wavelength and no applied voltage (Note S5, Supporting Information) confirmed that noise from laser wavelength and intensity fluctuations is negligible compared to modulation‐induced noise. When using a single MZI or MRR, the mean and standard deviation of device weights are constrained to follow a fixed functional relationship, as shown by the fitted lines in Figure 2c‐ii,f‐ii. This constraint limits the flexibility in generating tunable probabilistic distributions. By placing the MZI before the MRR, the combined PRNG enables tuning of *μ* and *σ* across a 2D surface, providing access to all *μ*‐*σ* combinations within the tunable range. Figure 2g‐i illustrates independent control of *μ_out_* with constant *σ_out_* by the PRNG. Here, the MRR voltage is held at 0 V to maintain *σ_out_* at ≈0.04 V, while the MZI voltage modulates *μ_out_*. Figure 2g‐ii presents a heatmap of *μ_out_* under various MZI and MRR voltage combinations, showing trends consistent with Figure 2c,f. Independent control of *σ_out_* with stable *μ_out_* is shown in Figure 2h‐i. Adjusting the MRR voltage significantly alters *σ_out_*, while the MZI voltage only slightly impacts it, as shown in Figure 2h‐ii. Maintaining *μ_out_* stability requires concurrent MZI voltage adjustments, because MRR changes both μ and σ simultaneously.

The proposed PRNG structure, leveraging MZI and MRR spectral characteristics, is adaptable to devices with varying parameters. A second PRNG design based on another MRR also achieved independent *μ*‐*σ* control (Note S6, Supporting Information). Furthermore, add‐drop MRR structure is also a feasible alternative for constructing the PRNG, highlighting the versatility of the design.

## 3 Robust Image Classification by PBNN

**Figure 3:**
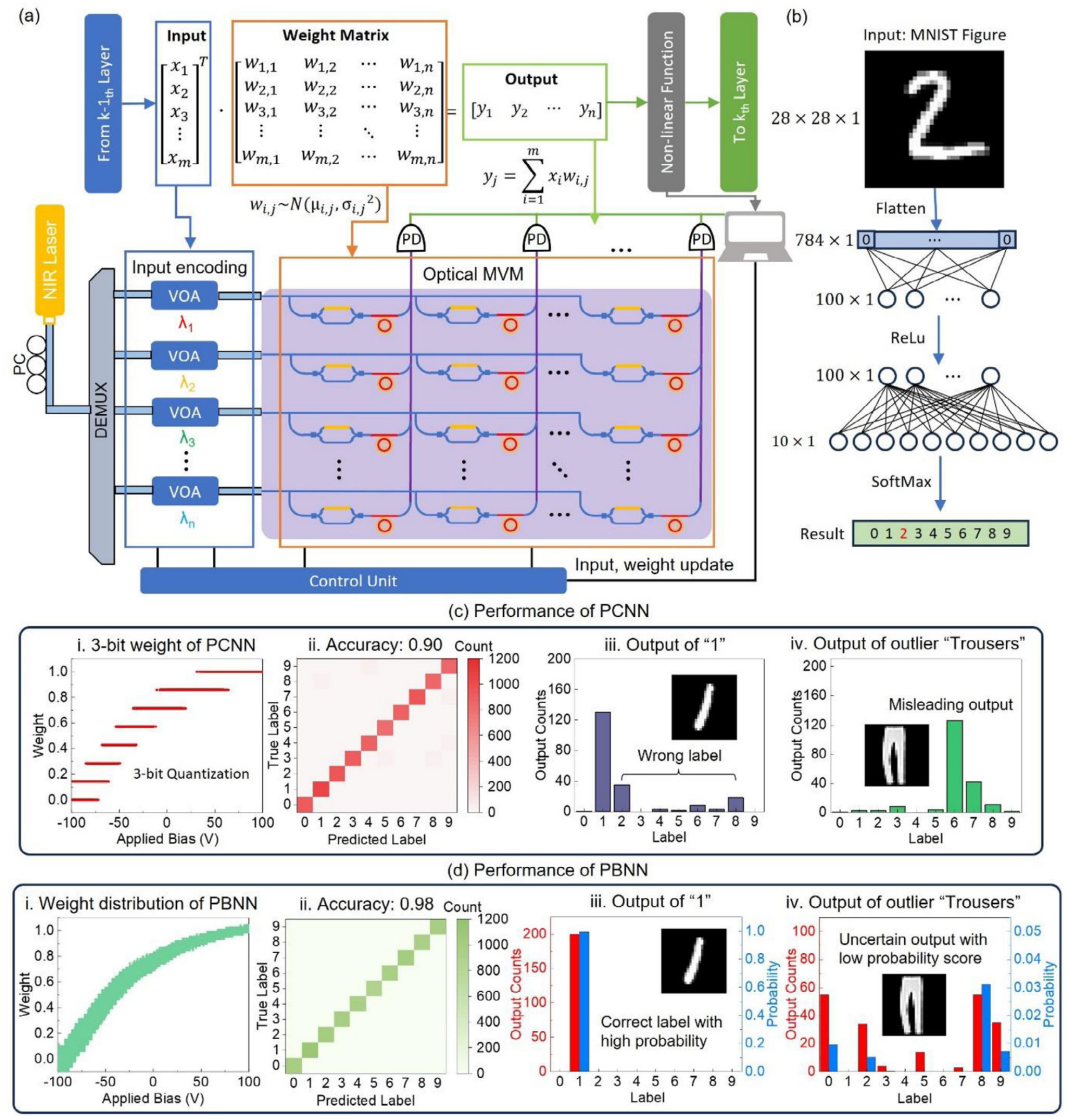
MNIST digit recognition using PBNN and PCNN. a) Schematic of PBNN implementation and architecture. b) Network structure for MNIST digit recognition. c) Performance of PCNN. i) 3‐bit weights in PCNN using PRNG data from Figure 2. Specific applied biases can produce multiple weight values due to noise. ii) Confusion matrix for 3‐bit PCNN on the MNIST task. Accuracy decreases from 0.98 to 0.90 because of noise impact. iii) Output of digit “1” for PCNN (200 samples). iv. Output of outlier ("Trousers") for PCNN (200 samples). The PCNN produces a high‐frequency output for both expected and unexpected inputs, and its predictions are unstable due to the influence of device noise. d) Performance of PBNN. i) Weight distribution of PBNN using the same PRNG data as in c. ii) Confusion matrix for PBNN on the MNIST task. Accuracy remains comparable to a full‐precision neural network, even with noise impact. iii) Output of digit "1" for PBNN (200 samples). All outputs correctly identify the label, with high probability scores close to 1, indicating reliability. iv. Output of outlier ("Trousers") for PBNN (200 samples). Outputs are ambiguous, with low probability scores close to 0, reflecting low reliability.

Using the proposed PRNG as the building block of BNN layers, various PBNNs can be constructed for different tasks. **Figure** **3**a illustrates the basic operations within the hidden layers of a PBNN, where the output of the (k‐1)^th^ layer serves as the input to the k^th^ layer. This input undergoes MVM with the weight matrix. Following the MVM operation, the output is processed by a nonlinear activation function, which produces the final output of the layer and feeds it to the subsequent layers. The key distinction between BNNs and deterministic neural networks lies in the weight matrix. In deterministic networks, the weights are fixed high‐precision values learned during continuously fitting the input‐output relationships. In contrast, in BNNs, the elements of the weight matrix are probabilistic distributions, allowing the network to model uncertainty. This probabilistic approach provides PBNNs with enhanced robustness and the ability to quantify uncertainty, making them particularly well‐suited for tasks involving complex or uncertain data. The lower part of Figure 3a illustrates the entire process of implementing the PBNN on a photonic platform, as well as the corresponding relationships between different elements of the neural network and experimental devices. The input vector is first converted into optical intensities at different wavelengths, which are separated into various channels from a multi‐wavelength laser source using a demultiplexer (DEMUX) and then encoded by variable optical attenuators (VOAs).

After encoding, the light from different channels is directed into on‐chip photonic multiply‐accumulate (MAC) units composed of the proposed PRNGs. These PRNGs are arranged in a crossbar array structure, where each PRNG functions as a distributed weight in the weight matrix. The crossbar array is a widely used architecture in photonic computing for performing large‐scale on‐chip matrix multiplication. Its primary function is to uniformly distribute the optical input signals across each array cell, where each cell performs an individual dot‐product operation with the input. The sum of the output intensities along each column corresponds to the result of MAC operations. Figure 3a shows a schematic of the PRNG array, and a detailed explanation of the operating principles and design of the crossbar array can be found in Note S7 (Supporting Information). The outputs of the MAC unit are detected by photodetectors and sent to an electronic unit for further processing, including data storage, distribution analysis, and nonlinear functions. Theoretically, the scalability of the PBNN can be enhanced for ultra‐compact AI applications by integrating more channels for parallel computing or reusing the same unit sequentially. Moreover, in BNNs, reusing the same device across different subnetworks allows for multiple samplings from the device characteristic, which theoretically improves the reliability of the neural network—an approach known as ensembled subnetworks.^[^ ^40^ ^]^

To demonstrate the advantages of the PBNN, we compare its performance with that of a PCNN, which uses traditional deterministic models, employing the same network structure for the Modified National Institute of Standards and Technology (MNIST) dataset classification task. The dataflow and network architecture are depicted in Figure 3b. The 28×28 pixel, 8‐bit grayscale images are flattened and fed into the hidden layer as network input. We use the measured results of the PRNG from Figure 2 as weights in the neural networks. When using the PCNN with fixed‐valued weights, as shown in Figure 3c‐i, device noise cannot be ignored. Even when the weight is quantized to 3‐bit precision, mitigating the noise impact is challenging, particularly when the target weight value is small, it becomes challenging to identify a specific applied voltage that maps uniquely to single weight level. As a result, the final accuracy of the 3‐bit PCNN reached only 0.9, significantly lower than the ideal accuracy of 0.98. For relatively basic classification tasks like MNIST, a 3‐bit classical neural network model already provides sufficient parameter space to capture the features of the dataset. Under ideal (noise‐free) conditions, such a model can achieve accuracy close to that of a full‐precision model, reaching ≈98%. However, in PCNNs, the presence of noise from photonic devices reduces the actual performance, limiting the accuracy to ≈90%.

Using higher‐bit quantization does not significantly improve performance in this scenario, as the increased expressiveness of high‐bit models cannot be fully utilized for simple tasks like MNIST. On the contrary, the fine‐tuned weights in high‐bit models tend to be more sensitive to noise, which further contributes to accuracy degradation. As shown in Note S8 (Supporting Information), device noise negatively impacts PCNN performance across different quantization levels. Both high‐bit and low‐bit models (except for extremely low‐bit models like 1 or 2 bits, which inherently struggle to reach acceptable accuracy) experience accuracy loss due to noise, confirming that device noise is a key limiting factor for PCNN performance.

Additionally, 0.9 represents the average accuracy across multiple PCNN inferences, with output instability being another major issue. Deterministic models are prone to overfitting, where even minor perturbations in weight values can lead to significant fluctuations in final output, despite identical input data. Figure 3c–iii shows 200 sampling results of the PCNN's output when classifying the digit “1”. Although many outputs correctly correspond to label 1, ≈70 of the 200 samples produced incorrect labels. While selecting the most frequent label as the final output is a possible solution, this introduces a new issue: detecting unexpected inputs (outliers). The PCNN, trained on the MNIST dataset, still produces distributed outputs with a specific high‐frequency label when exposed to unexpected inputs, such as images from the Fashion‐MNIST dataset (Figure 3c–iv). It is difficult to distinguish between expected and unexpected inputs based on the output distributions shown in Figure 3c‐iii,c‐iv. Note S9 (Supporting Information) shows more PCNN outputs under different expected and unexpected inputs, which all have the same issue. This issue arises from the nature of deterministic model training, which is essentially a maximum likelihood estimation process aimed at finding the maximum *P*(*D*|*W*). Deterministic models only focus on finding weights, *W*, that maximize the probability of the desired output, *D*. As a result, the uncertainty of data *P*(*D*) and the uncertainty of weights *P*(*W*) are not considered in this approach. Exactly because deterministic models cannot handle uncertainty in either data or weights, the output uncertainty observed in PCNNs is merely an external manifestation of weight uncertainty caused by device noise. In an ideal, noise‐free scenario, such models produce fixed, overconfident outputs—consistently predicting the same label for both expected and unexpected inputs, regardless of their similarity to the training data. When hardware noise is present, as in photonic implementations, this noise introduces fluctuations around the overfitted output, but these variations simply reflect instability in the physical system, not learned uncertainty. The output variability thus cannot be interpreted as meaningful uncertainty estimation. Further discussion and supporting evidence are provided in Note S10 (Supporting Information).

In contrast, the training process of a BNN can be regarded as a maximum posterior estimation, with the objective of maximizing *P*(*W*|*D*). By applying Bayes' rule, *P*(*W*|*D*) can be expressed as: *P*(*W*|*D*)  =  *P*(*D*|*W*)*P*(*W*)/*P*(*D*). This formulation includes both the uncertainty of the weights and the dataset, making the model more statistically robust. For the MNIST inference in the PBNN, we use continuous, distributed outputs of the PRNG as Bayesian weights, as shown in Figure 3d‐i. The distribution of each weight element in the PBNN is pre‐trained on a PC and then applied to the corresponding PRNG unit. The confusion matrix in Figure 3d‐ii demonstrates the high accuracy of the PBNN (0.98), which is nearly equivalent to the ideal accuracy of a full‐precision neural network on a PC.

Unlike deterministic networks, where the output is ideally fixed for the same input,^[^ ^41^, ^42^ ^]^ the output of a BNN can be interpreted as a probability model *P*(*y*∣*D*, *W*), which corresponds to the SoftMax output score in the case of classification. Consequently, the SoftMax output score of a BNN is typically referred to as a probability score, indicating the network's confidence in the result. Both the probability score and the distribution of the PBNN's output reflect the reliability of the result. Figure 3d‐iii shows the PBNN's output for the same input image of the digit “1”. Unlike the PCNN, the PBNN's output labels are consistently correct, with all 200 samples accurately pointing to label “1”. The blue bar in Figure 3d‐iii represents the average probability score, which is nearly 1, indicating the PBNN's high confidence in the result. For other predicted expected inputs, the PBNN also provides consistent outputs with high probability scores (Note S11, Supporting Information). The PBNN exhibits markedly different behavior when processing unexpected inputs. Figure 3d‐iv shows the output distribution of the PBNN for the same outlier “Trousers” image from the Fashion‐MNIST dataset. Since the PBNN has learned the data distribution and uncertainty characteristics of MNIST dataset, it responds with high uncertainty when presented with the outlier, which shares very little similarity with the training set. In this case, the network produces a wide range of predicted labels with significant output variability, yet all associated with probability scores close to zero, indicating the PBNN's low confidence and high uncertainty in its predictions for such unfamiliar inputs.

This behavior highlights the PBNN's ability to quantify uncertainty: for inputs that diverge significantly from the training distribution, the model avoids overconfident decisions. By analyzing both the probability scores and the output distribution, one can easily distinguish expected inputs from outliers—something that is not achievable with PCNNs. The PBNN's performance in outlier detection demonstrates its effective integration of device noise to model weight uncertainty, enabling it to capture and represent intrinsic data uncertainty through a robust probabilistic framework.

## 4 Multimodal Gesture Recognition by PBNN

**Figure 4:**
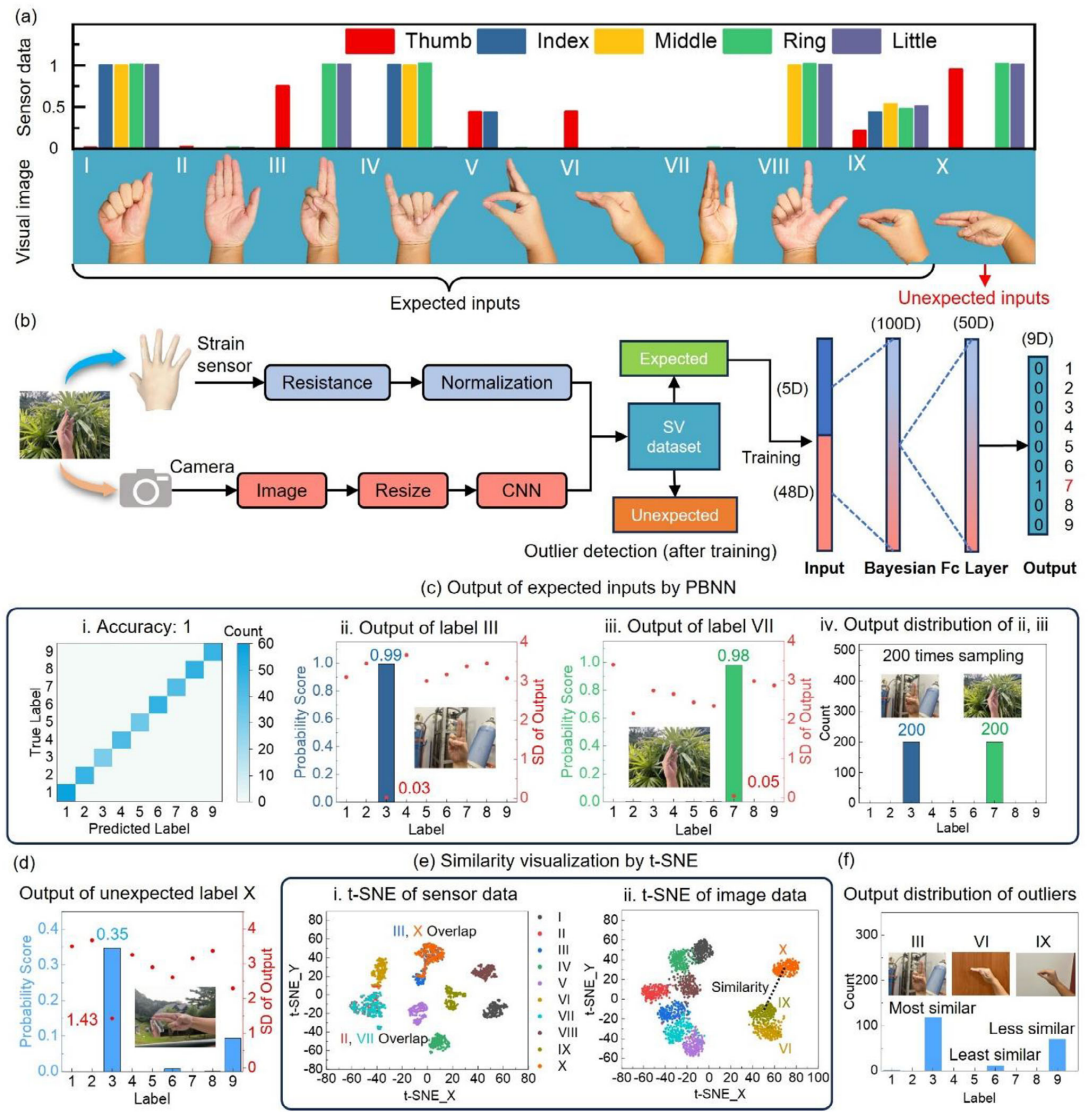
Gesture recognition using the PBNN. a) Schematic of the SV dataset. The first 9 gestures are treated as expected inputs for training, while the 10th gesture is reserved as an unexpected input. b) Data collection, processing, and fusion in PBNN. The combined 48D visual data and 5D sensor data fused through 2 Bayesian fully connected (Fc) layers in the PBNN. c) Performance for expected inputs. i) Confusion matrix showing almost 100% accuracy for expected inputs. ii) Output for label III. iii) Output for label VII. iv) Output distribution for labels III and VII. All 200 samples provide correct results. d) Performance for unexpected input (label X). e) Similarity visualization via 2D t‐SNE. i) t‐SNE of sensor data. ii) t‐SNE of image data. f) Output distribution for unexpected input (label X). The output is predominantly assigned to labels with similarity to label X, with label III receiving the highest count due to its close similarity. Output counts decrease as similarity diminishes.

In the era of fifth generation mobile networks and the Internet of Things, a vast number of sensors are required to monitor the physical states of various objects. By integrating data from multiple sensors, multimodal datasets are created, providing a more comprehensive description of object states.^[^ ^43^ ^]^ However, the fusion of datasets with varying spans and dimensions introduces greater uncertainty and increases the processing complexity for deterministic classical neural networks, which are more susceptible to overfitting on such complex datasets. In this part, we used the somatosensory–visual (SV) dataset, which fuses visual images with somatosensory data from skin‐like electronic devices for human gesture recognition.^[^ ^33^ ^]^ The SV dataset consists of 3000 samples distributed across 10 categories of hand gestures. Each sample includes an image of a hand gesture captured against a complex background and a set of strain data recorded from five strain sensors placed over the knuckles of each finger. The gestures and corresponding sensor data for each category are shown in **Figure** **4**a. For feature extraction, the image data is compressed into a 48D vector using an AlexNet convolutional neural network, which provides a cost‐effective and energy‐efficient visual representation of each gesture. The 5D strain data, collected from resistive sensors, is normalized and then combined with the processed image data to form the SV dataset, which serves as the input for the PBNN.

It can be clearly observed that there is no significant difference between gestures III and X, aside from the direction, resulting in almost identical sensor data. Similarly, although gestures II and VII differ in appearance, neither involves significant finger bending, leading to minimal sensor data output for both. When examining the image data, gestures VI, IX, and X share visual similarities, which could lead to misjudgement under challenging conditions such as complex backgrounds or poor lighting. While the PCNN struggles to handle such a dataset with high uncertainty, the PBNN not only achieves high accuracy but also facilitates outlier detection and similarity analysis.

To demonstrate these advantages, we selected label X as the unexpected input, while the other gestures were treated as expected inputs. The data collection and network processing workflow are depicted in Figure 4b. Figure 4c shows the performance of the PBNN on expected inputs. Since the PBNN accounts for the uncertainty in the data, it handles the complex SV dataset effectively, achieving nearly 100% accuracy. Figure 4c‐ii,c‐iii illustrate the average and standard deviation of the PBNN's output probability score for label III and VII inputs, respectively. To more intuitively illustrate the differences in output standard deviation across labels, the figure presents the standard deviation of the logarithm of the probability scores. The PBNN output was sampled 200 times for each input data. For these expected inputs, the PBNN produced a high probability score with a low standard deviation on the correct label. For incorrect labels, the probability scores were close to zero but had relatively higher standard deviations, indicating the network's uncertainty and lack of trust in these outputs (more examples can be found in Note S12, Supporting Information). All results of the 200 samplings confidently point to the correct label, as shown in Figure 4c‐iv.

In contrast, for the unexpected input label X, the PBNN exhibits high uncertainty, with a large standard deviation for each label, which is significantly larger than that of the expected inputs. Additionally, the probability scores of all labels are much lower than 100%, with only labels III, VI, and IX showing relatively higher probability scores, as illustrated in Figure 4d. We can also observe that the probability score is inversely related to the standard deviation; labels with higher probability scores consistently have lower standard deviations, indicating a higher level of confidence. The PBNN does not randomly select these three labels as preferred outputs for the outlier but conducts label selection based on data similarity. Figure 4e shows the processed image and sensor data visualized using the t‐distributed stochastic neighbor embedding (t‐SNE) method, which is employed for dimensionality reduction and visualization.^[^ ^44^ ^]^ Points corresponding to the same gesture category (represented by the same color) cluster together, forming ≈10 categories of hand gestures (I to X). The overlap in some categories is due to similarity. For instance, the finger bending states in labels III and X are almost identical, making them difficult for sensors to distinguish. A similar case occurs between labels II and VII. In the image data, label X's cluster is relatively close to labels IX and VI, with IX being the closest to X, indicating greater similarity. According to the t‐SNE analysis, the similarity between labels III and X is the highest, followed by IX, and VI is the least similar one. The output of the PBNN mirrors this trend, as shown in Figure 4f: among the 200 samples, the PBNN outputs are most biased toward label III, followed by IX, and finally VI. These results demonstrate the PBNN's ability to handle complex multimodal datasets with high robustness and to indicate similarities, which holds potential in fields like medicine. For example, some diseases may mutate based on the original condition, and a BNN could effectively avoid misdiagnosis by identifying possible sources of the mutation and its similarities to the original disease. This probabilistic approach not only aids in accurate diagnosis but also provides valuable insights for advancing neural network development and addressing uncertainty‐driven challenges across critical domains.

## 5 Robust Spectrum Regression by PBNN

In addition to the classification tasks discussed previously, where the neural network produces discrete outputs by establishing decision boundaries between classes, the PBNN also performs effectively in regression tasks. In these tasks, the network learns a continuous function to model the relationship between input features and target values. The statistical nature of the PBNN allows it to represent this relationship not as a fixed function but as a dynamic interval that incorporates the uncertainty inherent in the data and the outputs. Here we performed mid‐infrared (MIR) spectroscopic data processing for a regression task.^[^ ^45^ ^]^ Due to the abundant molecular absorption peaks in the MIR wavelength range, photonic waveguide sensors provide advantages for label‐free and non‐destructive sensing.^[^ ^46^, ^47^, ^48^, ^49^ ^]^ However, neural network algorithms are still essential for handling complex environments and target mixtures.^[^ ^50^ ^]^

**Figure 5:**
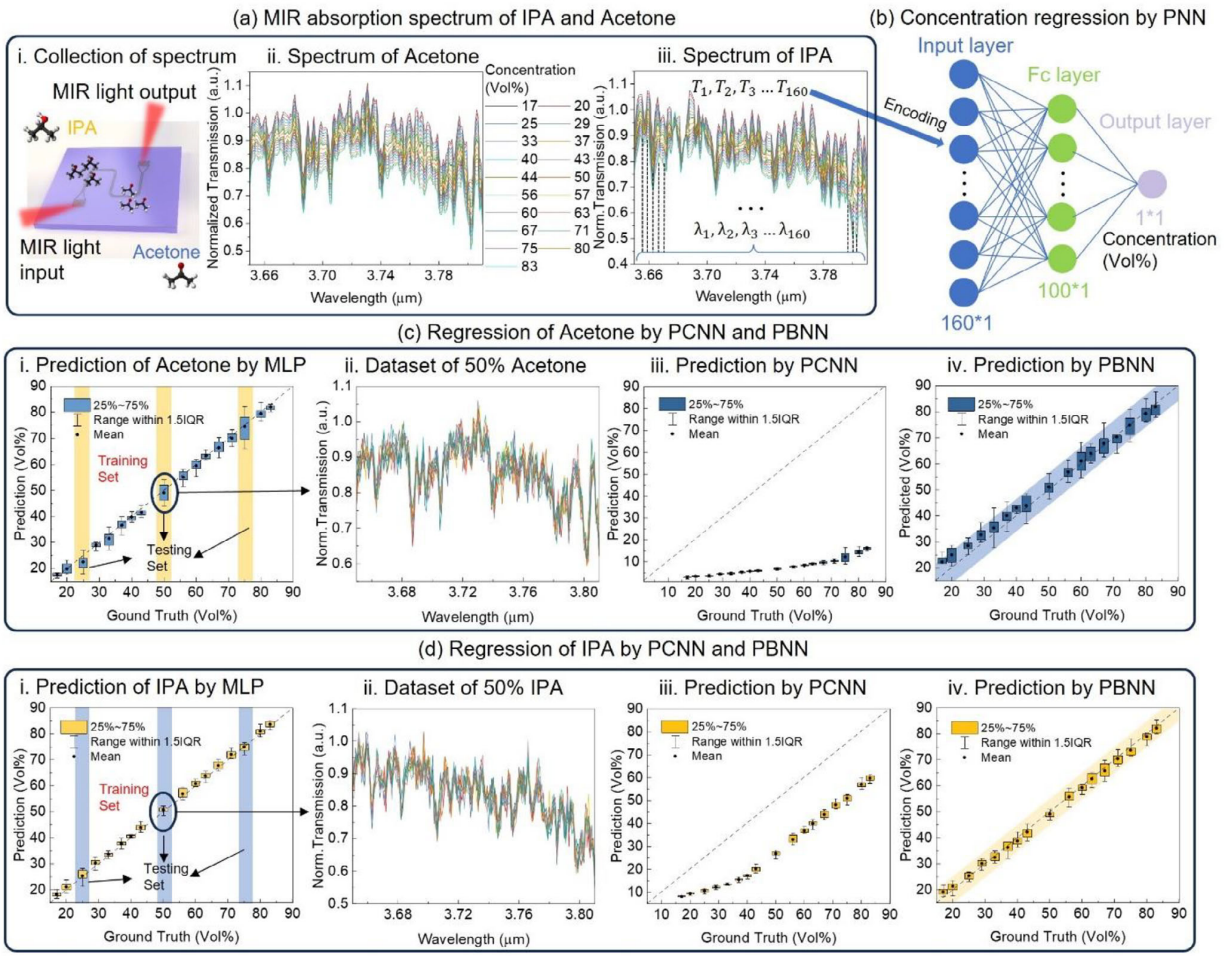
Gas spectrum regression using PCNN and PBNN. a) MIR absorption spectrum of IPA and Acetone. i) Schematic of spectrum data collection, where IPA and Acetone gases flow over the chip separately to obtain their pure absorption spectra. ii) Fitted absorption spectra of Acetone at varying concentrations. iii) Fitted absorption spectra of IPA at varying concentrations. b) PNN architecture for concentration regression. c) Regression results for Acetone using PCNN and PBNN. i) Acetone concentration prediction using MLP without noise impact. Among 19 concentrations, 25%, 50%, and 75% are used for testing, while the others are used for training. ii) Testing data for 50% Acetone, showing fluctuations caused by noise during measurement. iii) Acetone concentration prediction using a PCNN. Pre‐trained weights affected by device noise cause significant degradation in prediction accuracy. iv) Acetone concentration prediction using a PBNN. Pre‐trained Bayesian weights make predictions robust to noise and provide confidence intervals to represent input data uncertainty. d) Regression results for IPA using PCNN and PBNN i) IPA concentration prediction using MLP without noise impact. ii) Testing data for 50% IPA, showing smaller fluctuations compared to Acetone. iii) IPA concentration prediction using a PCNN. iv) IPA concentration prediction using a PBNN. The smaller fluctuation in IPA input spectra results in higher confidence predictions, as indicated by narrower confidence intervals.

We used spectroscopic data measured from 19 different concentrations of pure isopropyl alcohol (IPA) and acetone using an MIR waveguide sensor. For each concentration, the spectrum was measured 10 times, and these measurements were combined to form the dataset. **Figure** **5**a‐ii,a‐iii show the averaged spectrum for each concentration, which displays good linearity consisting with the absorption principle. We extracted the normalized transmission values at 160 equally spaced wavelengths as input for the PBNN. 16 of the 19 concentrations were used for training, while the remaining concentrations (25%, 50%, and 75%) served as test sets.

Traditionally, regression tasks are tackled using MLPs, a form of classical neural networks. If this task were performed entirely on a personal computer, the MLP would be capable of handling the task and providing high prediction accuracy, as shown in Figure 5c‐i,d‐i. However, for different spectra of the same concentration, the network is unable to produce identical output concentrations. This is due to environmental disturbances and noise during measurement, which can cause slight variations even for the same chemical and concentration. Figure 5c‐ii,d‐ii show the measured spectra for 50% acetone and 50% IPA across 10 samples respectively, with an average spectrum noise standard deviation of ≈0.03.

When the weight values of the MLP are transferred to the optical devices of the PNN, the prediction accuracy significantly decreases due to the impact of noise. Figure 5c‐iii,d‐iii show the performance of PCNN with a normalized device noise level of 0.03, which is derived from the MZI results in Figure 2. The normalized noise level represents the ratio of output noise to output range, which for the MZI is ≈0.03 (calculated as 0.016V/(0.73V–0.17 V)). Although the PCNN predictions deviate notably from the actual values, the output fluctuation for the same concentration is even smaller, meaning it no longer reflects input data uncertainty.

For comparison, we trained a PBNN on the same task, and the prediction results are shown in Figure 5c‐iv,d‐iv. The PBNN achieves relatively high accuracy and is robust to device noise. Additionally, unlike traditional MLPs, where the output is fixed for identical inputs, the PBNN produces outputs that follow a distribution, corresponding to the uncertainty of the dataset. The shaded areas in Figure 5c‐iv,d‐iv represent the PBNN's output confidence intervals. The standard deviation of the PBNN's output for the IPA dataset averages ≈2%, while for acetone it is ≈3.5%. This output uncertainty aligns with the uncertainty in the input data, derived by a statistical analysis of spectrum intensity fluctuations. For acetone, the average standard deviation is 0.03, which is 0.002 higher than that of IPA. While this difference may seem small, Figure 5a shows that the spectral differences between different concentrations are minimal, meaning even slight perturbations could lead to misjudgements in network output. Unlike the deterministic PCNN, the PBNN not only analyses the relationship between chemical concentration and output spectrum but also accounts for uncertainties caused by experimental errors, providing a confidence interval to avoid overfitting.

## 6 Conclusion

In this work, we present a PBNN built upon a novel PRNG that harnesses intrinsic photonic noise within the photonic system. Unlike deterministic PCNNs, which are vulnerable to device noise and fluctuations, our PBNN not only mitigates the impact of noise but also utilizes it to manage data uncertainty, establishing a foundation for robust probabilistic photonic computing.

By capitalizing on the analog nature of optical signals, the PBNN overcomes the limitations of electronic probabilistic processors, which rely on cycle‐to‐cycle variations as random sources and face challenges such as sequential sampling, limited endurance, and slow switching times.^[^ ^28^, ^29^, ^30^, ^51^, ^52^ ^]^ Our design uniquely employs output uncertainty caused by limited precision in the driving voltage, eliminating dependence on ASE and accommodating hardware constraints. Using the spectral properties of MZIs and MRRs, the PBNN achieves flexible control over output distributions without being strictly bound by device parameters.

While PRNG has not yet been integrated into a large‐scale photonic MAC unit, this can be achieved using a crossbar array architecture combined with directional couplers for power allocation.^[^ ^9^ ^]^ This configuration would allow scalable MVM operations with low energy consumption and high computational throughput. Simulations confirm the PBNN's high predictive accuracy, noise resilience, and ability to detect unexpected inputs and perform probabilistic predictions. These features, absent in deterministic models, are crucial for applications like medical diagnostics and autonomous driving, where data uncertainty and limited training data pose significant challenges. As AI evolves to require systems capable of evaluating multiple outcomes rather than deterministic responses, our PBNN offers a promising path forward, equipping photonic systems to address the increasing complexity and speed demands of future AI applications.

## 7 Experimental Section

### Fabrication of AlN/Si Platform

The PRNG was designed and fabricated on a bilayer AlN/Si waveguide platform using the advanced 8‐inch photonic foundry facilities. The fabrication process begins with a standard SOI wafer featuring a 220 nm device layer, forming the foundation for subsequent chip component definitions. Deep ultraviolet (DUV) lithography and precise silicon etching were used to define the Si waveguide. Next, a SiO_2_ layer was grown via plasma‐enhanced chemical vapor deposition (PECVD), utilizing Tetraethyl orthosilicate (TEOS) as the precursor. Chemical mechanical planarization (CMP) was then applied to create a flat surface suitable for further processing.

Following this, AlN was deposited through physical vapor deposition (PVD). Another round of DUV lithography and etching defines the AlN waveguide, aligning it with the Si waveguide to ensure efficient light coupling and modulation. TEOS‐based SiO_2_ deposition and CMP were then repeated to add insulating layers and maintain surface flatness. Additional DUV lithography and etching define the SiO_2_ pattern, creating the necessary height difference for the electrode arrangement. Finally, TiN and Al layers were sequentially deposited and etched to form the microheater for thermo‐optic devices and the electrodes, respectively, completing the fabrication process with precision.

### Characterization of Photonic Random Number Generator

The PRNG characterization was conducted using a customized near‐infrared (NIR) fiber‐optic alignment system. NIR light from a tunable laser (Keysight 81960A) was coupled into NIR optical fibers (Thorlabs SMF‐28). A 6‐axis manual alignment stage (Kouzu GXM07S) was used to align the chip with the optical fibers, ensuring efficient coupling of NIR light into the photonic waveguide and transmission to the output fiber. The output spectrum was measured by a power meter (Keysight 81636B), synchronized with the tunable laser source.

In the spectrum scan method, a fixed DC voltage of 20 V, generated by a tunable DC power supply (Agilent E3631A), was applied to the devices under test via a ground‐signal‐ground (GSG) probe (MPI T26A GSG100). The spectrum of MRR is scanned 500 times with a minimum tuning step of 2 pm, covering a wavelength range from 1577.25 to 1577.37 nm. For the voltage scan method, a Keithley 4200A‐SCS Parameter Analyzer generates the scanning voltage, applied through a GSG probe. The optical output measured by the power meter is connected to an oscilloscope (Agilent DSO‐X3034A) via a BNC cable for signal monitoring. For the voltage scan measurement of MZI, the input laser wavelength at 1577.29 nm while scanning the voltage from 0 to 8 V in 0.05 V intervals for 200 rounds was maintained. The input laser power was fixed at ≈10 dBm. For the voltage scan of MRR, the input laser wavelength was also maintained at 1577.29 nm, and the voltage was scanned from −100 to 100 V in 5 V intervals for 600 rounds.

### Training and Inference of Bayesian Neural Network

According to Bayes' theorem, the weights *W* of a BNN are defined by posterior probability distributions, as shown in Equation (1).

(1) $$p\left(W|D\right)=\frac{p\left(D|W\right)\cdot p\left(W\right)}{p\left(D\right)}$$

Here, *D* represents the training data, *p*(*W*|*D*) is the posterior distribution, and *p*(*D*|*W*) is the likelihood. *p*(*W*) denotes the prior, and *p*(*D*) is the evidence. The primary distinction between deterministic models and BNNs lies in their optimization objectives: deterministic models aim to maximize the likelihood, seeking to increase the probability of expected outputs for a given set of weights, while BNNs aim to maximize the posterior, focusing on identifying the most probable weights of the given data. Because Gaussian distributions are closed under operations such as addition and multiplication, assuming Gaussian forms for the prior *p*(*W*) and the likelihood *p*(*D*|*W*) often allows the posterior to be derived analytically in closed form. This eliminates the need for iterative methods or approximations which are computing intensive. Following many previous BNN‐related studies, this work similarly employs Gaussian distributions to approximate the prior distribution of weights.

However, because the evidence *p*(*D*) is usually computation intensive, the true posterior distribution *p*(*W*|*D*) in BNNs is generally intractable by directly calculating the Equation (1), methods such as variational inference and Markov Chain Monte Carlo (MCMC) sampling are employed to approximate it using an easily computable distribution. Variational inference was typically preferred for its superior convergence and scalability over MCMC.

In the variational inference method, *p*(*W*|*D*) is approximated using a combination of variational posterior distributions, typically Gaussian for convenience, denoted as $q_{\Phi }\left(W\right)$, where Φ represents the variational parameters. For a Gaussian distribution, these parameters include the mean μ and standard deviation σ. The approximation is achieved by minimizing the Kullback‐Leibler divergence between *p*(*W*|*D*) and $q_{\Phi }\left(W\right)$, which measures the dissimilarity between the two distributions. The Kullback‐Leibler divergence is expressed in Equation (2).

(2) $$\begin{array}{ccc} & & d_{KL}\left(q_{\Phi }\left(W\right)\;|\;p\left(W|D\right)\right)=E_{q_{\Phi }\left(W\right)}\left[\log q_{\Phi }\left(W\right)\right]-E_{q_{\Phi }\left(W\right)}\left[\log p\left(W|D\right)\right]\\ & & =E_{q_{\Phi }\left(W\right)}\left[\log q_{\Phi }\left(W\right)-\log p\left(W,D\right)\right]+\log p\left(D\right) \end{array}$$

The term log *p*(*D*) is independent of the parameters of $q_{\Phi }$ and can therefore be ignored during optimization. Consequently, the objective function can be reformulated to maximize the evidence lower bound $\tilde{L}\left(\Phi \right)$ (ELBO), as shown in Equation (3).

(3) $$\tilde{L}\left(\Phi \right)=E_{D,q_{\Phi }\left(W\right)}\left[\log p\left(W,D\right)-\log q_{\Phi }\left(W\right)\right]$$

During training, μ and σ for each synapse are optimized through standard backpropagation. Through backpropagation, the difference between $q_{\Phi }\left(W\right)$ and true posterior *p*(*W*|*D*) can be minimized to a negligible level. As a result, $q_{\Phi }\left(W\right)$ can be considered a good approximation of *p*(*W*|*D*), effectively capturing the weight distribution that characterizes the given dataset. In the inference phase, multiple forward passes were evaluated, with each Gaussian weight distribution sampled once per pass. Thus, a BNN can be considered as a collection of various deterministic neural networks, capturing a range of possible outcomes.

### Implementation of Photonic Bayesian Neural Networks for Different Tasks

For the image classification task, we used the MNIST dataset, which comprises 60 000 training images and 10 000 testing images. Both the PBNN and the PCNN utilized the same network architecture with a single hidden layer of 100 neurons and ReLU as the activation function. The final output was a 10‐element vector using the SoftMax scores for the 10 classes, corresponding to digits 0 through 9.

For the multimodal SV dataset classification task, the dataset was divided into 2400 training samples and 600 testing samples for the PBNN. The network architecture included two hidden layers: the first with 100 Bayesian neurons and the second with 50 Bayesian neurons, both using ReLU as the activation function. Since label X was designated as the outlier, the final output was a 9‐element vector using the SoftMax scores for the remaining 9 classes.

For the spectrum regression task, spectra for 19 concentrations were measured 10 times each, forming the dataset. Sixteen concentrations were used for training, while 25%, 50%, and 75% concentrations served as test sets. The PBNN featured a single hidden layer with 100 Bayesian neurons, used ReLU as the activation function, and directly predicted the concentration values.

### Statistical Analysis

In this study, both the outputs of photonic devices and the inference results of the neural networks are obtained through random sampling. Since the resulting distributions exhibit Gaussian‐like behavior, we performed Gaussian distribution fitting using Origin to extract the corresponding mean (μ) and standard deviation (σ) from the sampled histograms. These fitted parameters are used throughout the work to characterize the statistical properties of photonic weights and neural network outputs.

To maintain clarity and conciseness in the main manuscript, only a subset of representative output distributions is shown in the main text. However, we emphasize that all presented results are unbiased examples. The remaining distributions, although not displayed, exhibit consistent behavior and support the same conclusions—demonstrating the PBNN's superior performance in probabilistic analysis and noise robustness compared to PCNNs.

To provide a more comprehensive overview, additional output distributions for various input conditions have been included in Note S13, (Supporting Information). These results further validate that the statistical behavior observed and discussed in the main manuscript is representative and repeatable across different devices, inputs, and network configurations.

## Conflict of Interest

The authors declare no conflict of interest.

## Supporting information



Supporting Information

## Data Availability

The data that support the findings of this study are available from the corresponding author upon reasonable request.
