# Supplementary material for: Photonic Bayesian Neural Networks: Leveraging Programmable Noise for Robust and Uncertainty‐Aware Computing
Source: Adv Sci (Weinh). 2025 Apr 27;12(23):2500525. doi: 10.1002/advs.202500525 (PMC12199374; doi:10.1002/advs.202500525)
Supplement: Supplementary file 1 — Supporting Information [file ADVS-12-2500525-s001.docx]

Supporting Information

**Photonic Bayesian Neural Networks: Leveraging Programmable Noise for Robust and Uncertainty-Aware Computing**

*Yangyang Zhuge, Zhihao Ren, Zian Xiao, Zixuan Zhang, Xinmiao Liu, Weixin Liu, Siyu Xu, Chong Pei Ho, Nanxi Li*, and Chengkuo Lee**

Contents

[Note S1 Weight normalization and quantization. 2](#_Toc194182497)

[Note S2 Micro-ring resonator (MRR) and MZI of photonic random number generator (PRNG). 3](#_Toc194182498)

[Note S3 Output distribution of MZI and MRR 4](#_Toc194182499)

[Note S4 Simulation of spectrum shift induced by effective index fluctuation 5](#_Toc194182500)

[Note S5 Measurement of noise without applied voltage 7](#_Toc194182501)

[Note S6 PRNG implementation based on another MRR (R=50μm, Gap=0.35μm) 8](#_Toc194182502)

[Note S7 Design principles of the PRNG crossbar array 10](#_Toc194182503)

[Note S8 Accuracy degradation of deterministic neural network because of noise impact 13](#_Toc194182504)

[Note S9 Output distribution of photonic classical neural network (PCNN) on MNIST and F-MNIST inputs 15](#_Toc194182505)

[Note S10 PCNN’s performance difference under various noise levels 16](#_Toc194182506)

[Note S11 Output distribution of photonic Bayesian neural network (PBNN) on MNIST and F-MNIST inputs 18](#_Toc194182507)

[Note S12 Output probability score and standard deviation of PBNN on SV dataset expected inputs 19](#_Toc194182508)

[Note S13 Additional output distributions of PBNN 20](#_Toc194182509)

[Note S14 Difference between classical neural network and BNN 23](#_Toc194182510)

[Note S15 Comparison between PNNs harnessing noise for computing 24](#_Toc194182511)

Note S1 Weight normalization and quantization.

To convert the transfer function of photonic devices into the weight values used in neural networks, a normalization process is required. Here, we use the measured results of the Mach-Zehnder Interferometer (MZI) from Figure 2 as an example.
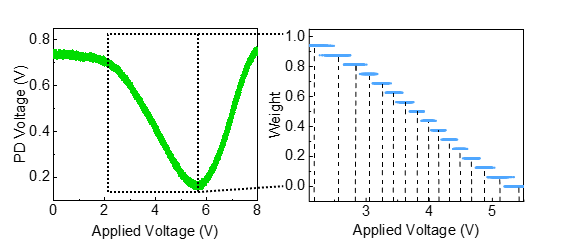


**Figure S1.** Transfer function of MZI and corresponding quantized weight values.

The left part of Figure S1 shows the transfer function of the balanced MZI from Figure 2, with an input power of 10 dBm. The applied voltage is selected within the range of approximately 2 to 6 V, which is sufficient to achieve a large modulation depth. The maximum output $V_{max}$ is about 0.73V and the minimμm output $V_{min}$ is about 0.17V. The normalized weight value $W$ corresponding to a specific output $V_{out}$ can be calculated using Equation (1):

$$\begin{aligned} W=\frac{V_{out}-V_{min}}{V_{max}-V_{min}}\#\left( 1 \right) \end{aligned}$$

Using Equation (1), the weights are normalized between 0 and 1, but they remain float numbers with an unavoidable noise level. In conventional photonic neural networks, quantization methods are commonly applied to mitigate noise effects.

The right part of **Figure S1** illustrates the 4-bit quantized weight values of the MZI. Uniform quantization is employed, dividing the weights into 16 equally spaced intervals between 0 and 1. The weight value of the n^th^ state is given by $\frac{n-1}{16}$.

Note S2 Micro-ring resonator (MRR) and MZI of photonic random number generator (PRNG).


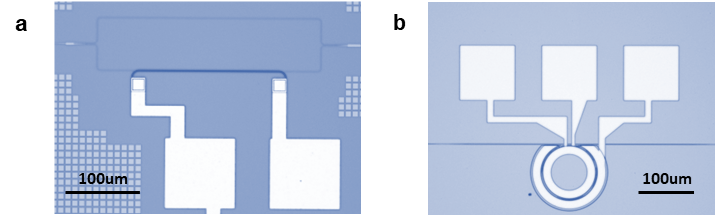


**Figure S2.** Optical microscopic image of MZI and MRR in the PRNG. (a) Image of the Silicon MZI. The length of both arms is 300μm. The width of Silicon waveguides is 0.45 μm. (b) Image of AlN/Si MRR. The radius of the ring is 50 μm. The gap between ring and bus waveguide is 0.4 μm. The width of AlN waveguides is 1 μm.

Note S3 Output distribution of MZI and MRR

In our study, the noise observed in the optical device outputs is primarily induced by electrical noise during the modulation process. This electrical noise—such as thermal noise and shot noise—exhibits an approximately Gaussian distribution in the time domain. This modulated signal with noise can lead to variations in the refractive index change of photonic devices, which can also be approximated as a Gaussian distribution in the time domain. As a result, the output distributions of our photonic devices, influenced by this modulation fluctuation, also approximate Gaussian distributions.

**Figure S3** illustrates the measured output distributions of the thermo-optic MZI and electro-optic MRR used in this work, sampled 200 times under their respective modulation conditions. These distributions can be well-fitted by Gaussian functions. In our analysis, both the output mean (μ) and standard deviation (σ) of the device outputs are extracted using Gaussian fitting of the measured output histograms.

Moreover, due to the closed-form mathematical properties of the Gaussian distribution, when the MZI and MRR are combined to form a PRNG, the resulting output—effectively a product of the two transmission functions—also follows an approximate Gaussian distribution.


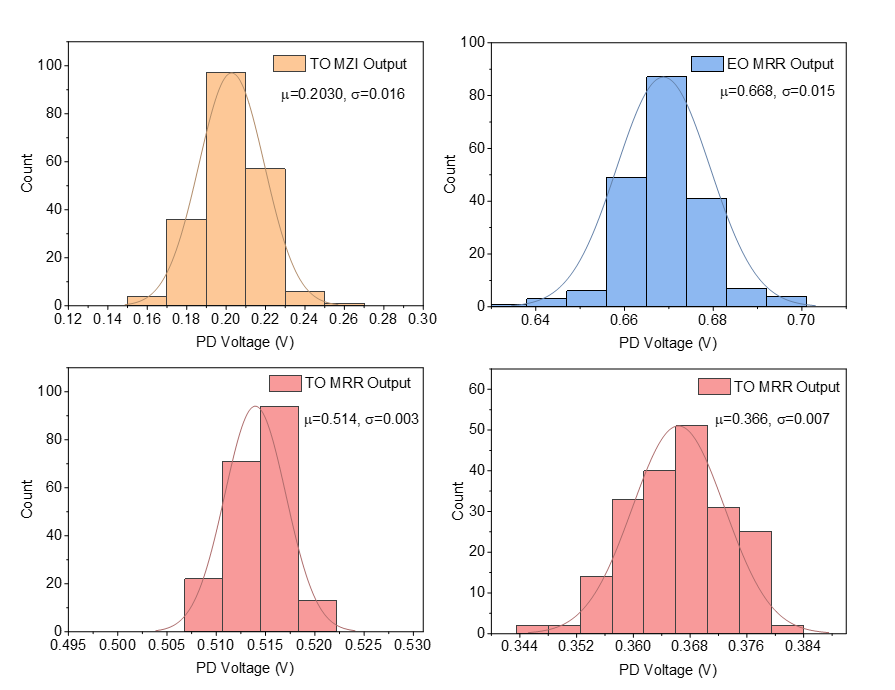


**Figure S3.** Output distributions of MZI and MRR in this work. The output influenced by electrical noise can be well fitted by Gaussian distributions.

Note S4 Simulation of spectrum shift induced by effective index fluctuation


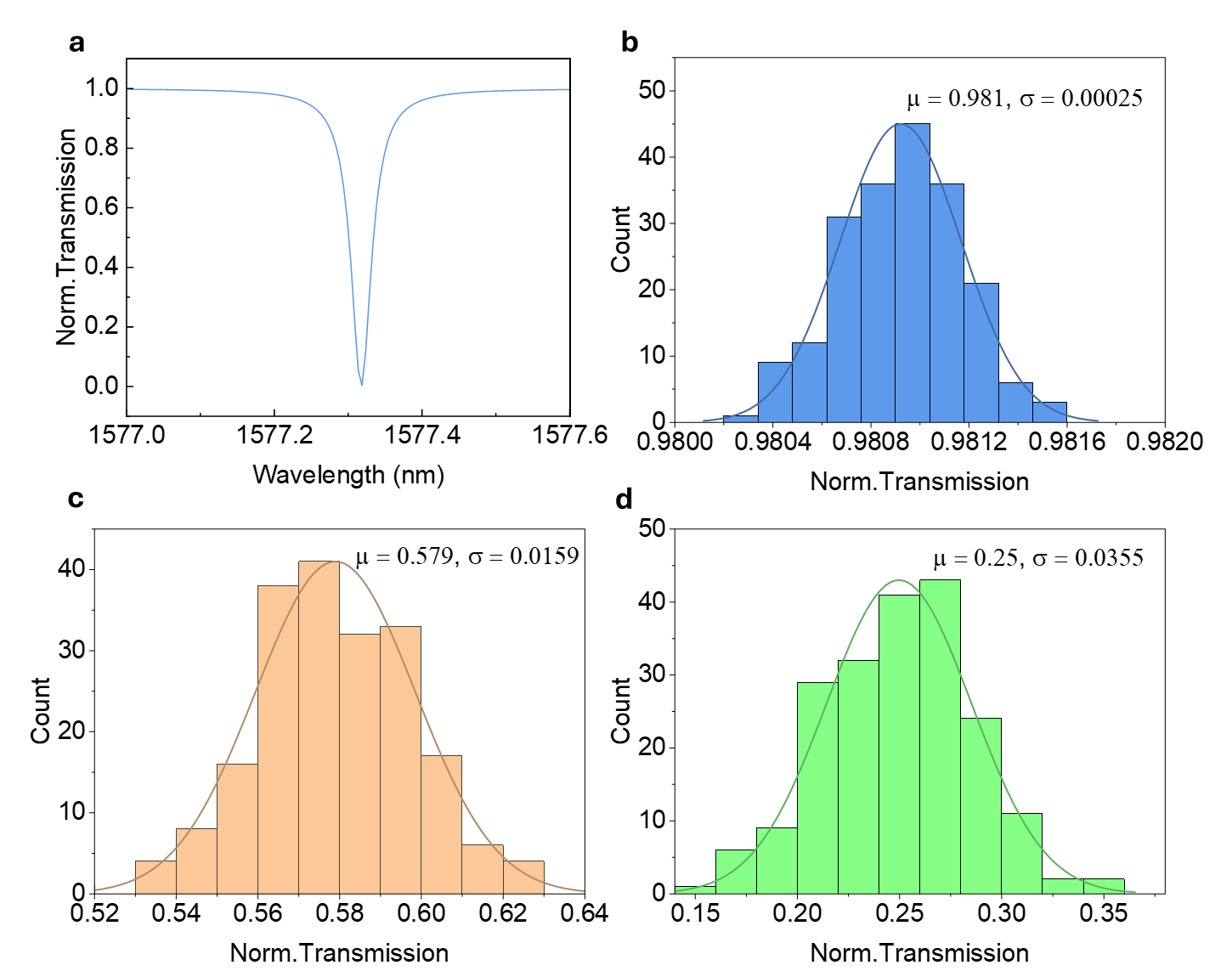


**Figure S4.** Simulation of modulation-induced noise of MRR. (a) Reconstructed MRR spectrum. (b), (c) and (d) Noise level of MRR output under various wavelengths, with same refractive index fluctuation.

We conducted simulations to verify the noise characteristics of the MRR. Based on the 20 dB extinction ratio and a quality factor of 45,000 from the MRR spectrum in Figure 2, the propagation loss was estimated to be approximately 1 dB/cm using Equation (2):$\begin{aligned} \alpha=\frac{\lambda*10\log\left( e \right)}{Q_{i}*r*FSR}\#\left( 2 \right) \end{aligned}$

Here, $Q_{i}$ the intrinsic quality factor, is approximated as twice the quality factor under conditions of large extinction ratio (critical coupling). With a ring radius of 50μm and a measured free spectral range (FSR) of 3.695, the group index $n_{g}$ was derived using Equation (3):

$$\begin{aligned} n_{g}=\frac{\lambda^{2}}{FSR*L}\#\left( 3 \right) \end{aligned}$$

Where $L$ is the round-trip length of MRR. The calculated group index is about 2.14, and the effective index of the AlN waveguide obtained via simulation, is about 1.706. Using these parameters, the spectrum was reconstructed with MATLAB simulations, as shown in **Figure S4(a).**

The wavelength noise in the MRR originates from effective index fluctuations caused by unstable applied voltages. The effective index change $\Delta n_{eff}$ is related to the resonance shift $\Delta\lambda_{res}$ by Equation (4):

$$\begin{aligned} \Delta n_{eff}=\frac{\Delta\lambda_{res}*n_{g}}{\lambda_{res}}\#\left( 4 \right) \end{aligned}$$

For a resonance peak tunability of 0.26 pm/V, the corresponding effective index change is about $3.544\times{10}^{-7}/V$. Considering the driving voltage noise level of 2V, the standard deviation of the effective index change is about $7\times{10}^{-7}$.

By incorporating this noise into the effective index, we simulated the spectrum shift induced by the noise. Sampling the transmission at different wavelengths near resonance allowed us to derive the output noise levels at various wavelengths, as shown in **Figure S4(b), (c), and (d).** The simulations reveal that noise levels increase as the output wavelength approaches the resonance wavelength, consistent with experimental results.

Note S5 Measurement of noise without applied voltage


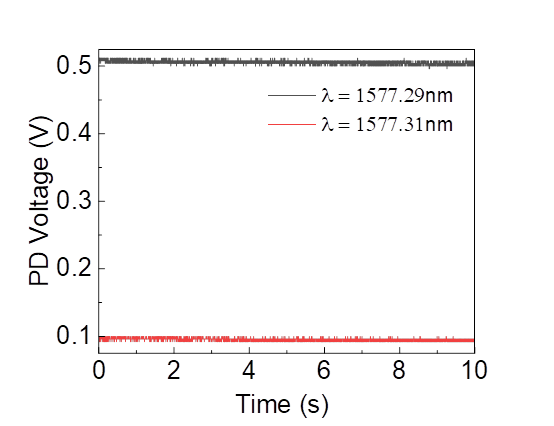


**Figure S5.** MRR Output of different wavelengths without applied voltage.

To measure wavelength noise from laser fluctuations and intensity noise from fiber alignment variations, we optimized fiber alignment in a stable testing environment and measured the output of the MRR without applied voltage. Two wavelengths (1577.29 nm and 1577.31 nm) were selected for testing. Although their output intensities differ significantly, the noise level remains nearly identical at approximately 0.002 V for both. Thus, wavelength and intensity noise in the absence of applied voltage are negligible compared to the electro-optical noise introduced by modulation voltage.

Note S6 PRNG implementation based on another MRR (R=50μm, Gap=0.35μm)


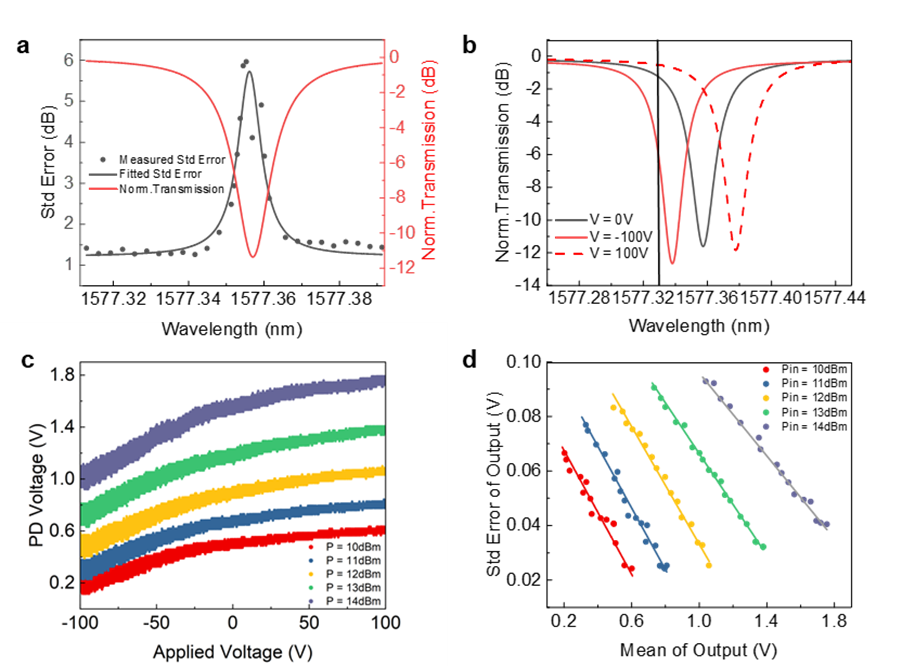


**Figure S6.** Uncertainty measurement of another all-pass MRR with different device parameters. (a) Uncertainty measured by spectrum scan. The applied voltage on the MRR is also 20V. (b) Voltage scan method for uncertainty measurement. The wavelength of input laser is fixed at 1577.33nm. The applied voltage scans from -100V to 100V. (c) MRR output with different applied voltage and input power. (d) Fitted mean and standard deviation of MRR output in (c).

To verify the universality of the method proposed in this article, we conducted uncertainty measurements on an additional all-pass MRR with different device parameters. In this device, the ring radius is 50 μm, and the gap between the ring and bus waveguide is 0.35 μm. Both the voltage scan and spectrum scan methods demonstrate the same output characteristics as those reported in the main text, specifically the inverse correlation between output light intensity and noise level.


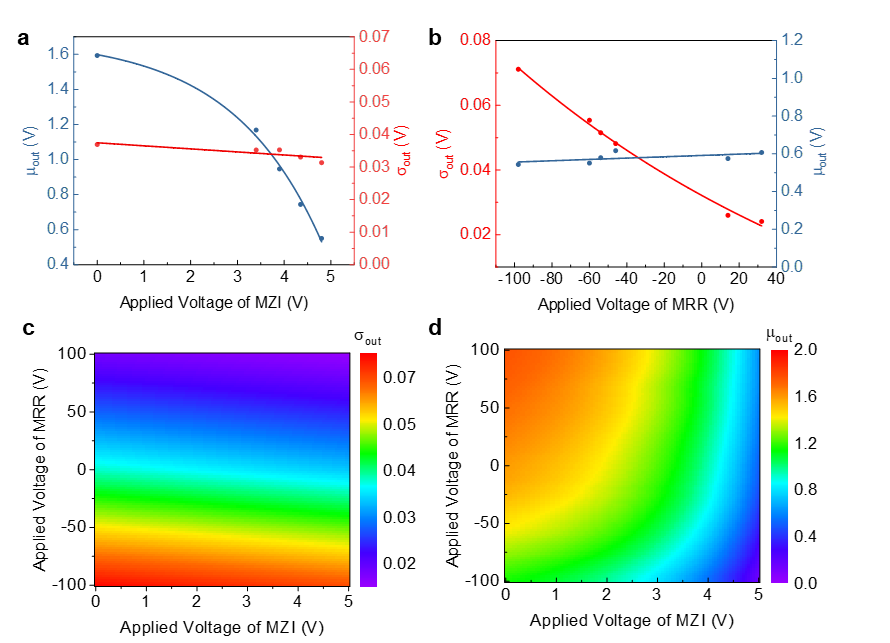


**Figure S7.** $\mu_{out}$ and $\sigma_{out}$ modulation of the PRNG based on another MRR. (a) $\mu_{out}$ independent modulation. (b) $\sigma_{out}$ independent modulation (c) Relationship between $\sigma_{out}$ and applied voltage on MRR and MZI (d) Relationship between $\mu_{out}$ and applied voltage on MRR and MZI.

A new PRNG can be realized by combining the MRR presented here with an MZI, allowing it to perform similar functions to the one described in the main text. Although the controllable ranges and values of μ and σ differ slightly between the two PRNGs, these variations can be managed by establishing lookup tables and setting parameters during network training.

Note S7 Design principles of the PRNG crossbar array

As the schematic in Figure 3 shows, the matrix used for probabilistic matrix-vector multiplication operations can be implemented using a crossbar array structure integrated with directional couplers, following the design principles.^[1]^ To provide a more detailed explanation of how this array architecture functions and how it can be scaled, we take a 2×2 array as an example to illustrate how the optical signal is evenly distributed to each PRNG cell for computation.


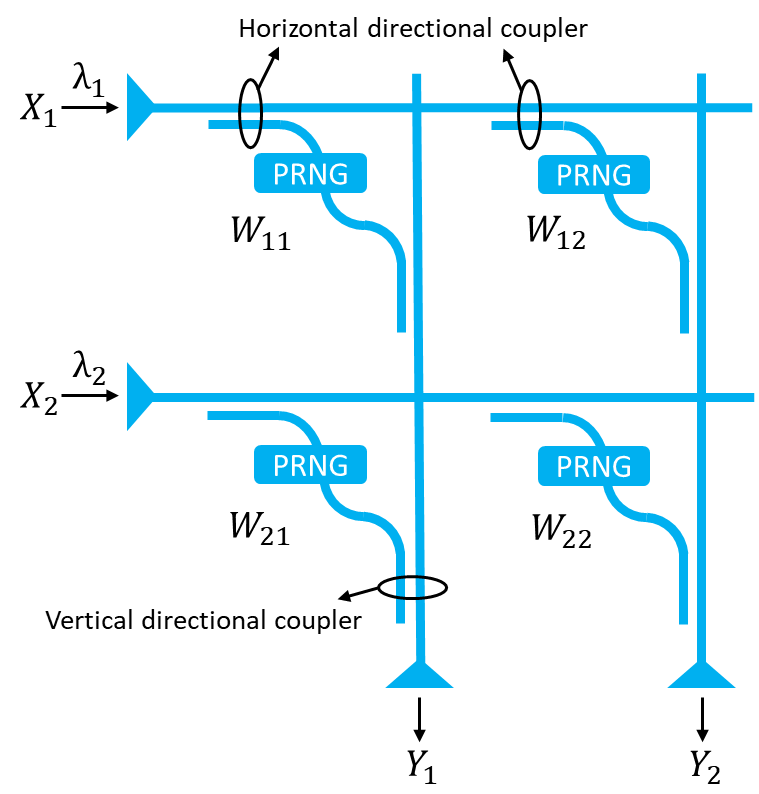


**Figure S8.** 2×2 PRNG crossbar array for probabilistic matrix-vector multiplication.

The horizontal directional couplers are responsible for distributing the input optical power equally across the matrix columns, while the vertical directional couplers collect the modulated signals from the PRNG cells and perform optical signal accumulation.

Each input vector entry interacts with only one PRNG cell per matrix column. This interaction represents a single multiplication between the optical input amplitude and the distributed weight encoded by the PRNG cell. The accumulated optical power at each column output is detected by a photodetector, representing the inner product of the input vector and the corresponding weight column. The resulting signal is scaled by a fixed factor of $1/(M \times N)$, where M and N denote the number of columns and rows in the matrix, respectively.

To prevent optical interference between the outputs of different PRNG cells in the same column—an issue that could degrade output accuracy—this work employs wavelength-division multiplexing (WDM). Specifically, incoherent light sources at different wavelengths are used for different rows in the PRNG array. This strategy ensures that the outputs from various PRNG cells remain independent and interference-free, allowing accurate detection of the output distribution by the column photodetectors.

Regarding the directional coupler design, the splitting ratios for the horizontal couplers are determined by the column index m and are given by $\frac{1}{M+1-m}$. With such splitting ratios, the split power for each column can be calculated as $\frac{1}{M}$. The splitting ratios for the vertical couplers can be calculated as $\frac{1}{n}$ with n being the number of the matrix row. Consequently, the optical power received at the output of column m from row n is: $\frac{1}{M}*\frac{1}{n}*\left( 1-\frac{1}{n+1} \right)*\left( 1-\frac{1}{n+2} \right)* \cdot\cdot\cdot*\left( 1-\frac{1}{N} \right)=\frac{1}{M*N}$, which is balanced across all PRNG cells.

The individual control of each device within the array can be achieved through the design of on-chip metal routing combined with off-chip control electronics. This is a well-established technique and has been widely implemented in most current photonic neural networks, enabling independent tuning of each photonic element on the chip.

Additionally, thanks to continuous advancements in photonic integrated circuit (PIC) technology, the optical loss of photonic devices has been significantly reduced, making the large-scale implementation of crossbar arrays increasingly feasible. For example, the largest demonstrated MRR crossbar array to date is a 16×16 array with an 800 µm pitch, as reported in *Nanophotonics* 12.20 (2023): 3883–3894.^[2]^

Regarding crosstalk, in the case of electro-optic (EO) modulation, the influence of the electric field from one device on its neighbours is minimal, allowing the crosstalk between devices to be safely neglected. In contrast, thermo-optic (TO) modulation introduces thermal crosstalk due to heat conduction when the spacing between devices is too small. This issue can be effectively mitigated by appropriately increasing the spacing between adjacent devices. For instance, in *Optica* 9.5 (2022): 579–584, ^[3]^ a 1×4 MRR array was demonstrated with a spacing of approximately 100 µm between the rings, achieving low thermal crosstalk and a high tuning accuracy of 9 bits through the use of a dithering control scheme.

Together, these strategies ensure the feasibility of scalable, low-crosstalk PRNG arrays suitable for integration in large-scale PBNNs.

Note S8 Accuracy degradation of deterministic neural network because of noise impact


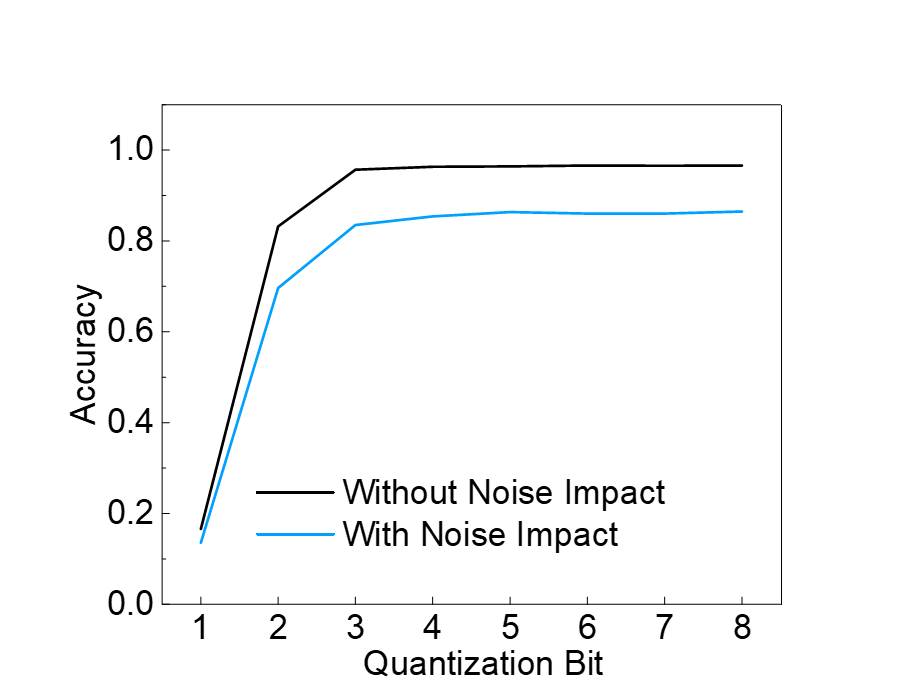


**Figure S9.** MNIST classification accuracy of classical deterministic neural network models with various quantization bits. The blue line shows the corresponding PCNN’s performance under the noise impact.

When conventional neural networks utilize higher quantization bits or full-precision floating-point representations for weights, the expressiveness of the parameter space increases, leading to improved model accuracy. While quantization can mitigate noise by mapping unstable outputs to discrete levels, overly coarse quantization—such as 1- or 2- bit representations—limits the model’s ability to capture complex features. As shown by the black curve in **Figure S9**, even in the absence of noise, low-bit quantized models exhibit reduced accuracy (below 0.9) on relatively simple tasks like MNIST classification. When the quantization level reaches 3 bits or more, the accuracy approaches that of full-precision models (~0.98), indicating sufficient expressive capacity for such tasks.

When noise impact is considered, it becomes challenging for conventional models to achieve ideal accuracy. The blue line in **Figure S9** illustrates the accuracy of quantized conventional models under noise impact. We use the μ-σ relationship of PRNG in Figure 2 to model the noise impact in PCNNs. High-bit models, which rely on precise parameters for feature characterization, are more sensitive to weight fluctuations. For example, the accuracy of an 8-bit high-precision model drops from 0.98 to 0.86 due to the noise impact. For low-quantization models, errors will still accumulate and influence the final output during matrix-vector multiplication. Moreover, low-precision models are inherently limited in feature extraction, and error accumulation exacerbates this issue. In the case of a 1-bit binary neural network, while the accumulated noise may not significantly alter its output state, its accuracy remains very low.

There are methods to improve the intrinsic accuracy of low-bit models, such as increasing the weight matrix size or introducing batch normalization layers with high-precision parameters. However, these approaches essentially compensate for the missing parameter dimensions in low-quantization networks. Thus, whether using high or low quantization models, significant accuracy loss occurs in PCNNs due to model precision limitations and noise influence.

Note S9 Output distribution of photonic classical neural network (PCNN) on MNIST and F-MNIST inputs


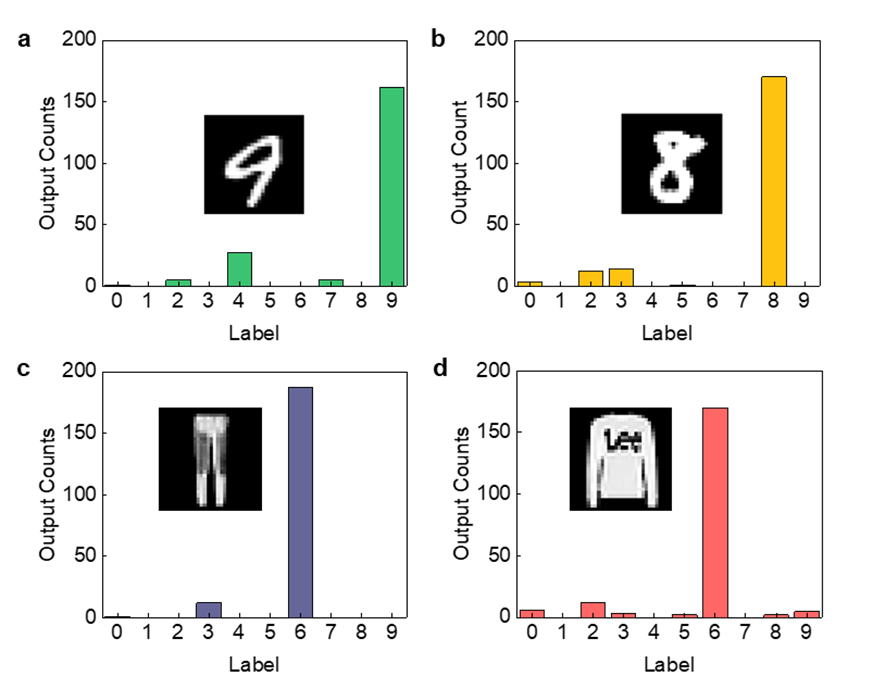


**Figure S10.** Output of photonic classical neural network. Here we consider the impact of noise. (a) Output distribution of input ‘9’. (b) Output distribution of input ‘8’. (c) Output distribution of outlier input ‘Trousers’. (d) Output distribution of outlier input ‘Pullover’.

This figure supplements the main text, showing the output distribution of the PCNN for both MNIST and F-MNIST inputs. Due to noise, the PCNN output becomes uncertain for any type of input. For MNIST (expected) inputs, the output with the highest frequency generally, though not always, corresponds to the correct label. However, for F-MNIST (unexpected) inputs, the PCNN’s output distribution resembles that of MNIST inputs, displaying uncertainty and a "confident" output with high frequency.

This similarity in output distribution makes it difficult to distinguish between outliers and expected inputs based on the PCNN’s output alone.

Note S10 PCNN’s performance difference under various noise levels


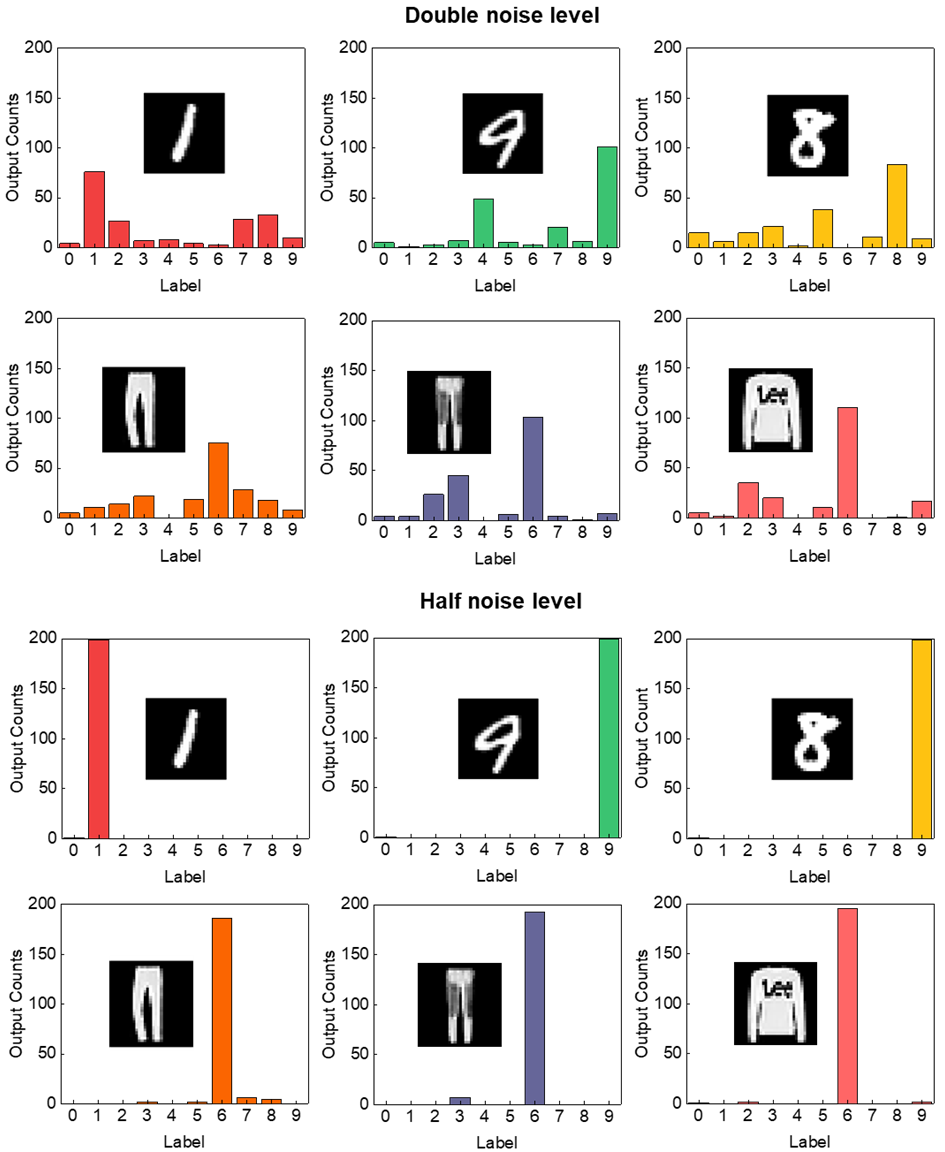


**Figure S11.** Output distribution of PCNN under various noise levels.

We further tested the PCNN under half and double the original noise level. Here, we also use the 3-bit quantized model from Figure 3, which achieves accuracy comparable to the full-precision model under ideal (noise-free) conditions. As shown in **Fig. S11**, with lower noise, classification accuracy increases to 95%, and the outputs are more concentrated, deviating less from the dominant label. In contrast, at double the noise level, outputs become more scattered, though still centered around the dominant class. This shows that noise in PCNN induces output variability, but does not reflect input uncertainty of the dataset. The deterministic nature of PCNN still leads to overconfident predictions for unseen data.

Note S11 Output distribution of photonic Bayesian neural network (PBNN) on MNIST and F-MNIST inputs


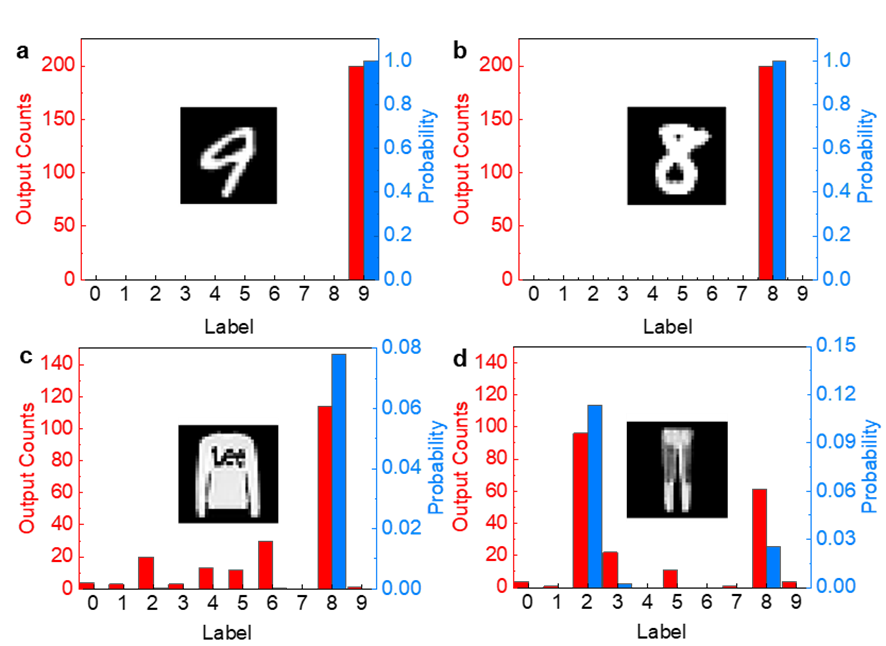


**Figure S12.** Output of PBNN. We also consider the impact of noise. (a) Output distribution of input ‘9’. (b) Output distribution of input ‘8’. (c) Output distribution of outlier input ‘Trousers’. (d) Output distribution of outlier input ‘Pullover’.

For the PBNN trained on the MNIST dataset, the outputs for MNIST inputs remain very stable across multiple samples, consistently pointing to the correct label. The probability score for the correct label is very high (close to 1), while the scores for other labels are extremely low (near 0).

In contrast, for F-MNIST inputs, the PBNN exhibits much greater uncertainty. Almost every label has a non-zero output frequency, but the probability score for each label remains quite low, typically below 0.2. This clear difference in output distribution enables easy identification of outliers by observing the PBNN’s responses.

Note S12 Output probability score and standard deviation of PBNN on SV dataset expected inputs


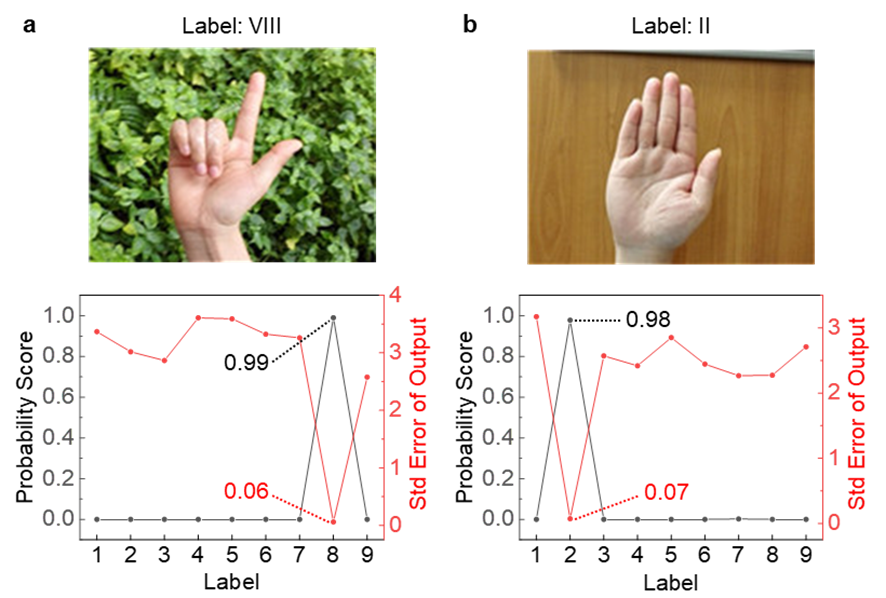


**Figure S13.** Output probability score and standard deviation of PBNN on SV dataset. (a) Output of label VIII. (b) Output of label II.

Here, we use the average probability score and fitted standard deviation of the PBNN’s output across 200 samples. For the SV dataset, the PBNN output consistently shows a high probability score (close to 1) for the correct label. Probability score and standard deviation are inversely correlated: outputs with higher probability scores have smaller standard deviations, and vice versa.

Note S13 Additional output distributions of PBNN

**Figure S14** shows representative output distributions for inputs of different classes from the MNIST dataset. As can be observed, for inputs that are visually distinct and easy to classify, nearly all 200 samples fall on the correct label, and the average output probability score for that label approaches 1.

Of course, the MNIST dataset also contains ambiguous or low-quality images. For instance, the second and third samples in the third row of the figure illustrate a "3" that resembles an "8" and a "5" that resembles a "6", respectively. In these cases, the PBNN occasionally outputs similar but incorrect labels during the 200 samplings—analogous to how a human might also misclassify such ambiguous images. We selected 200 samplings as it provides a sufficiently accurate representation of the output distribution. As a further validation, the final row of **Figure S14** shows the output distribution for the same inputs with the number of samplings increased to 400. The results confirm that the PBNN remains highly confident for expected inputs.

In contrast, for out-of-distribution inputs from the Fashion-MNIST dataset, the PBNN consistently produces highly uncertain and disordered output distributions. **Figure S15** illustrates several such examples, where the predicted probability scores are low and dispersed across multiple labels. This behavior is observed for both 200 and 400 sampling iterations, highlighting the PBNN’s ability to detect unfamiliar inputs and appropriately express low confidence and high uncertainty in its predictions.

**
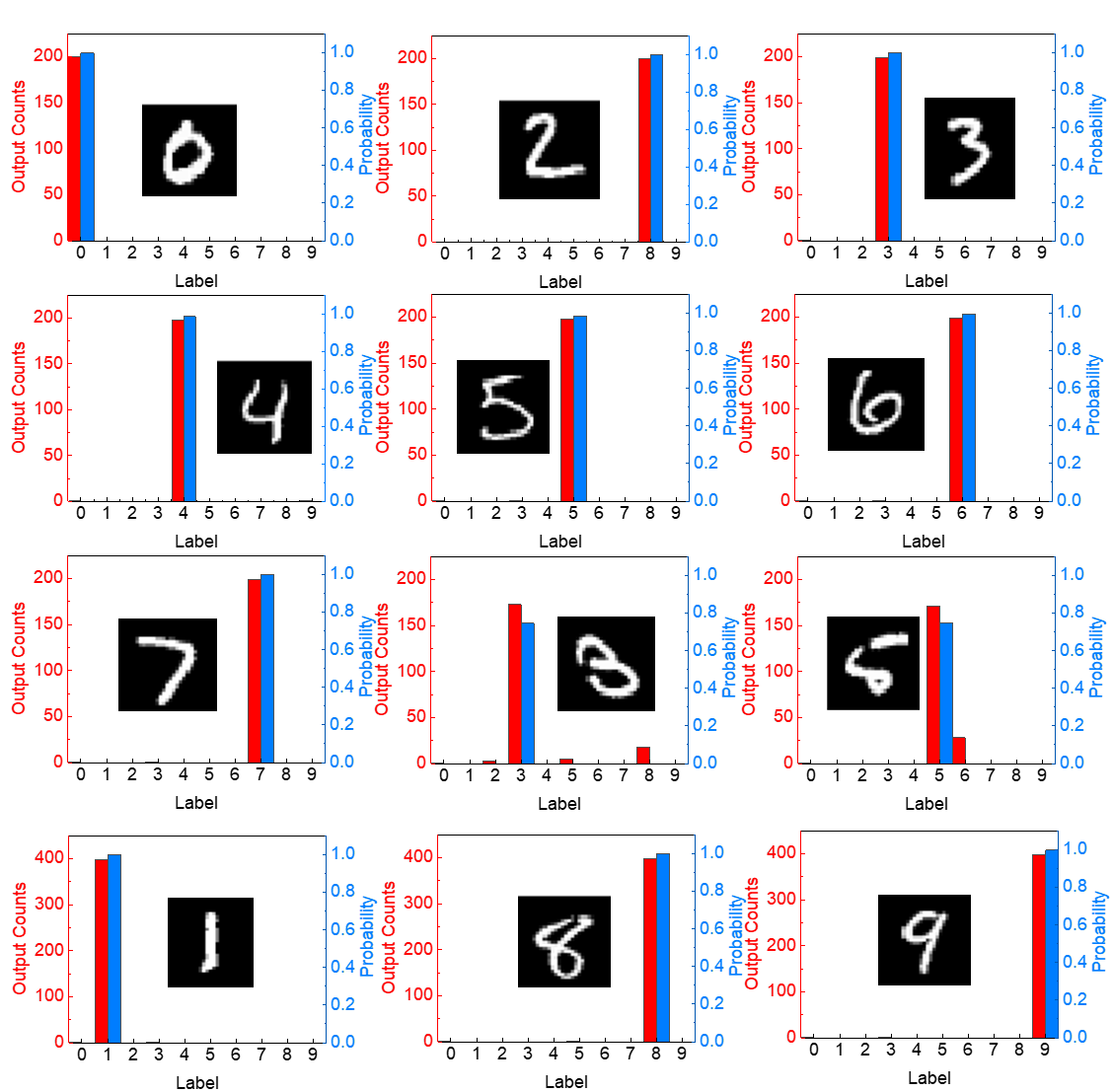
**

**Figure S14.** Output distributions of the PBNN with various input images. For clearly distinguishable inputs, the PBNN produces highly confident and accurate predictions. For ambiguous inputs, the network assigns the probable labels based on similarity to the training data, while also providing a corresponding probability that reflects this similarity. In this work, 200 sampling iterations were used, which are sufficient to capture the output distribution for each input image. Increasing the number of samples to 400 does not significantly alter the distribution, indicating the robustness and stability of the probabilistic predictions.

**
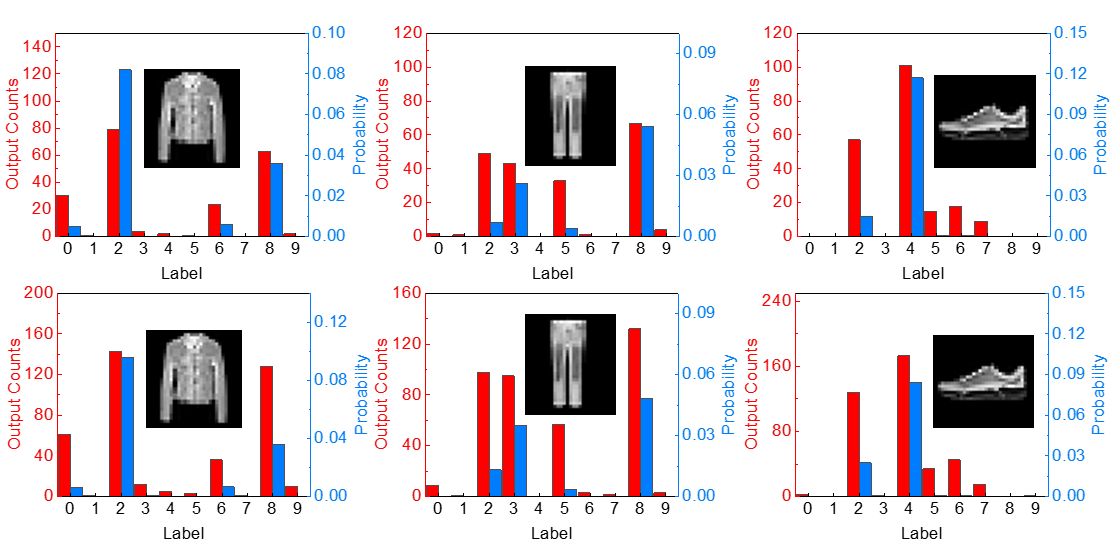
**

**Figure S15.** Output distributions of the PBNN with outlier inputs. Regardless of whether 200 or 400 samplings are performed, the PBNN consistently exhibits a dispersed and low-confidence output distribution for out-of-distribution inputs.

Note S14 Difference between classical neural network and BNN

|  | Classical neural network | | Bayesian neural network | |
| --- | --- | --- | --- | --- |
| Optimization | Maximize likelihood | | Maximize posterior | |
| Parameters (i.e. weight, bias) | Fixed value | | Probabilistic distribution | |
| Inference | Single run and choose class with largest output value | | Sampling distribution of output probability score | |
| Capability of probabilistic computing | No | | Yes | |
| Hardware platform | Electronic | Photonic | Electronic | Photonic |
| Principle | Conductivity modulation of electronic devices^[4]^ | Transmission modulation of photonic devices^[5]^ | Randomness in the switching process of memristors^[6]^ | Analog nature of optical signal^[7]^ |
| Speed | High | Ultra-high | Low | High |
| Energy consumption | High | Low | High | Low |

**Supplementary Table 1.** The difference between the classical neural network and BNN. Electronic and photonic implementations are compared in terms of principle and efficiency. The probabilistic computing capability in BNNs involves a trade-off, slightly sacrificing speed for enhanced uncertainty modelling and robustness.

Note S15 Comparison between PNNs harnessing noise for computing

|  | PBNN using programmed optical noise^[8]^ | Photonic generative network harnessing opto-electronic noise^[9]^ | Probabilistic photonic computing with chaotic light^[7]^ | This work |
| --- | --- | --- | --- | --- |
| Random source | Ampliﬁed spontaneous emission (ASE) | ASE | ASE | Modulation-induced photonic noise |
| Distribution tunability | Yes | No | Yes | Yes |
| Distribution modulation method | Separate modulation of μ and σ by MRR | N.A. | Adding up of ASE pulses with programmed intensities | Combined modulation of μ and σ by MRR and MZI |
| Experiment validation | No | Yes | Yes | Yes |
| Capability of probabilistic computing | Yes | No | Yes | Yes |
| Network operation of photonic parts | Probabilistic fully-connected layer | Deterministic fully-connected layer | Probabilistic convolution layer | Probabilistic fully-connected layer |
| Scalability | High | High | Low | High |

**Supplementary Table 2.** Comparison of related works on PNNs utilizing noise for computing: principles and performance. Our work achieves a more flexible and scalable modulation of weight distribution in the PNN.

Both referenced works about PBNN utilize the signal–ASE beat noise to generate random distributions, which can be described by the following expression:

$\sigma_{sig-ASE}^{2}=\frac{2R^{2}P_{sig}P_{ASE}}{M}$, where R is the responsivity of the photodetector, $P_{sig}$ and

$P_{ASE}$ are the optical signal power and the time-averaging ASE noise power, and the M is a conﬁguration constant: $M=\frac{2B_{O}}{B_{LP}}$, determined by the optical ﬁlter bandwidth $B_{O}$ and the photodetector bandwidth $B_{LP}$. From the formula describing signal–ASE beat noise, it can be observed that the noise level can be modulated by adjusting the optical signal power, assuming other factors such as ASE noise power remain constant. However, in this case, the mean and standard deviation of the output remain inherently correlated. To address this, both Wu *et al.*^[8]^ and Frank *et al.*^[7]^ employ fractional-step methods to separate the control of these two parameters.

Wu *et al.* compute the mean and standard deviation in two distinct steps. For mean calculation, their setup resembles a conventional MRR-based PNN, where the ASE source is not introduced. The mean value of the weight is modulated by tuning the MRR transmission at the input wavelength. For standard deviation computation, the optical signal is combined with an ASE source, and the MRR modulates the optical signal power to achieve the desired noise level. The mean component of this signal is filtered out using a DC block, leaving only the noise component. The final weight distribution is obtained by combining the results from both stages. While effective in theory, this approach requires distinct optical paths and configurations for the mean and standard deviation, as well as additional ASE sources and filtering components. This adds complexity to the system architecture and computational process. Furthermore, although Wu *et al.* demonstrated that ASE noise level varies with signal intensity, they did not experimentally validate the separate computation of mean and standard deviation using MRRs. Therefore, device-level issues such as noise modulation and signal crosstalk are not fully accounted for, potentially impacting accuracy in practical implementations.

Frank *et al.*, on the other hand, split each input into nine sub-symbols and modulate the aggregate distribution. According to the signal–ASE beat noise model, the standard deviation is proportional to the square root of the signal power. Thus, for the same total power, dividing the input into multiple symbols reduces the noise per symbol, enabling modulation of the overall distribution by adjusting individual intensities. This strategy effectively decouples the output noise from the total optical intensity, allowing independent control of the output variance. However, because the randomness is encoded and modulated in the input signal itself, the optical phase-change memory (PCM) devices in the array serve only as static weights. This limits scalability, as enhancing uncertainty resolution requires more optical input channels. Additionally, since the output distribution is derived by summing nine consecutive symbols, the need for multiple samplings constrains the inference speed and reduces precision. As a result, this architecture is more suitable for convolutional layers, where kernel reuse and smaller weight dimensions make this sampling approach more practical.

In contrast, our work introduces a compact, integrated PRNG architecture based on the combination of an MZI and MRR, which achieves independent and continuous control of both mean (μ) and standard deviation (σ) within a single modulation step. This design significantly simplifies the computational architecture of the PBNN while improving scalability. Because the PRNGs can be arranged in an array to implement distributed weight matrices, the proposed PBNN supports large-scale probabilistic MVM operations, enabling it to serve not only as a convolutional layer but also as a fully connected layer. This enhanced flexibility and integration make our approach more suitable for broader, scalable applications in photonic probabilistic computing.

**Reference**

[1] J. Feldmann, N. Youngblood, M. Karpov, H. Gehring, X. Li, M. Stappers, M. Le Gallo, X. Fu, A. Lukashchuk, A. S. Raja, J. Liu, C. D. Wright, A. Sebastian, T. J. Kippenberg, W. H. P. Pernice, H. Bhaskaran, Parallel convolutional processing using an integrated photonic tensor core, *Nature* **2021**, *589*, 52.

[2] J. Cheng, Y. Xie, Y. Liu, J. Song, X. Liu, Z. He, W. Zhang, X. Han, H. Zhou, K. Zhou, H. Zhou, J. Dong, X. Zhang, Human emotion recognition with a microcomb-enabled integrated optical neural network, *Nanophotonics* **2023**, *12*, 3883.

[3] W. Zhang, C. Huang, H.-T. Peng, S. Bilodeau, A. Jha, E. Blow, T. F. de Lima, B. J. Shastri, P. Prucnal, Silicon microring synapses enable photonic deep learning beyond 9-bit precision, *Optica* **2022**, *9*, 579.

[4] K.-U. Demasius, A. Kirschen, S. Parkin, Energy-efficient memcapacitor devices for neuromorphic computing, *Nat. Electron.* **2021**, *4*, 748.

[5] S. Ohno, R. Tang, K. Toprasertpong, S. Takagi, M. Takenaka, Si microring resonator crossbar array for on-chip inference and training of the optical neural network, *ACS Photonics* **2022**, *9*, 2614.

[6] A. Sebastian, R. Pendurthi, A. Kozhakhmetov, N. Trainor, J. A. Robinson, J. M. Redwing, S. Das, Two-dimensional materials-based probabilistic synapses and reconfigurable neurons for measuring inference uncertainty using Bayesian neural networks, *Nat. Commun.* **2022**, *13*, 6139.

[7] F. Brückerhoff-Plückelmann, H. Borras, B. Klein, A. Varri, M. Becker, J. Dijkstra, M. Brückerhoff, C. D. Wright, M. Salinga, H. Bhaskaran, B. Risse, H. Fröning, W. Pernice, Probabilistic photonic computing with chaotic light, *Nat. Commun.* **2024**, *15*, 10445.

[8] C. Wu, X. Yang, Y. Chen, M. Li, Photonic bayesian neural network using programmed optical noises, *IEEE J. Sel. Top. Quantum Electron.* **2022**, *29*, 1.

[9] C. Wu, X. Yang, H. Yu, R. Peng, I. Takeuchi, Y. Chen, M. Li, Harnessing optoelectronic noises in a photonic generative network, *Sci. Adv.* **2022**, *8*, 2956.
